# Quantitative Assessment of Tetrel Bonding Utilizing Vibrational Spectroscopy

**DOI:** 10.3390/molecules23112763

**Published:** 2018-10-25

**Authors:** Daniel Sethio, Vytor Oliveira, Elfi Kraka

**Affiliations:** Computational and Theoretical Chemistry Group, Department of Chemistry, Southern Methodist University, 3215 Daniel Avenue, Dallas, TX 75275-0314, USA; sethio.daniel@gmail.com (D.S.); vytor3@gmail.com (V.O.)

**Keywords:** noncovalent interactions, weak interactions, tetrel bonding, intrinsic bond strength, local stretching force constant, CCSD(T)

## Abstract

A set of 35 representative neutral and charged tetrel complexes was investigated with the objective of finding the factors that influence the strength of tetrel bonding involving single bonded C, Si, and Ge donors and double bonded C or Si donors. For the first time, we introduced an intrinsic bond strength measure for tetrel bonding, derived from calculated vibrational spectroscopy data obtained at the CCSD(T)/aug-cc-pVTZ level of theory and used this measure to rationalize and order the tetrel bonds. Our study revealed that the strength of tetrel bonds is affected by several factors, such as the magnitude of the *σ*-hole in the tetrel atom, the negative electrostatic potential at the lone pair of the tetrel-acceptor, the positive charge at the peripheral hydrogen of the tetrel-donor, the exchange-repulsion between the lone pair orbitals of the peripheral atoms of the tetrel-donor and the heteroatom of the tetrel-acceptor, and the stabilization brought about by electron delocalization. Thus, focusing on just one or two of these factors, in particular, the *σ*-hole description can only lead to an incomplete picture. Tetrel bonding covers a range of −1.4 to −26 kcal/mol, which can be strengthened by substituting the peripheral ligands with electron-withdrawing substituents and by positively charged tetrel-donors or negatively charged tetrel-acceptors.

## 1. Introduction

Noncovalent interactions (NCIs) have received increasing attention in the last two decades [1,2,3] due to their technological and fundamental importance in physics, chemistry, and biology [4,5,6]. Despite of the fact that NCIs are weak compared to covalent bonds (about an order of magnitude smaller), the importance of NCIs absolutely cannot be neglected [7,8,9]. They are ubiquitous and play a significant role in determining the properties of matter from small molecules to supramolecular systems like DNA and proteins [7,10]. They stabilize molecular structures [11,12], construct supramolecular materials [13], lower the activation energy of chemical reactions [14], and regulate the properties of crystal materials [15]. A series of different types of NCIs has been reported, namely, hydrogen bonds [16,17,18,19,20,21,22], aerogen bonds (group 18) [23,24,25,26], halogen bonds (Group 17) [27,28,29,30,31,32], chalcogen bonds (Group 16) [33,34,35,36,37], pnicogen bonds (Group 15) [38,39,40,41,42], tetrel bonds (Group 14) [43,44,45,46,47,48,49], and triel bonds (Group 13) [50,51].

**Figure 1:**
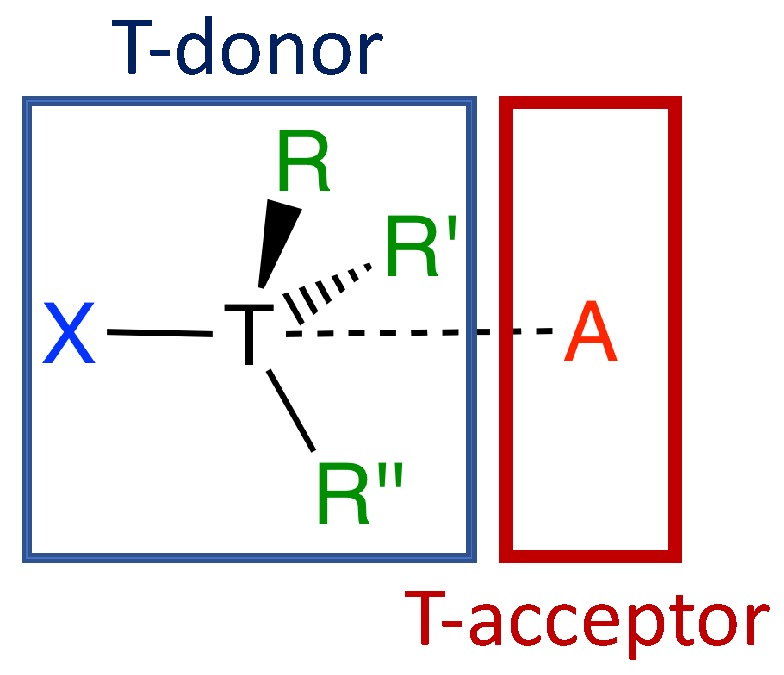
Schematic representation of tetrel complexes between the electron-deficient tetrel atom of a Lewis acid (tetrel donor, T-donor, T = C, Si, Ge) and the electron-rich tetrel atom of a Lewis base (tetrel acceptor, T-acceptor, A = FH, OH_2_, NH_3_, Cl^−^).

Recently, tetrel bonding has found many applications due to its unique properties, such as strength, directionality, and origin of attraction [52]. Tetrel bonds play an important role in crystal engineering and supramolecular chemistry as a new potential molecular linker [44,53] and in dynamical processes such as protein folding and ligand–acceptor interactions [54,55,56]. Tetrel bonds also play an important role in the preliminary stages of SN2 reactions [57] and hydrophobic interactions [58,59]. The formation of tetrel bonds can be understood as an interaction between an electron-deficient tetrel atom of a Lewis acid (tetrel donor, T-donor) and an electron-rich of a Lewis base (tetrel acceptor, T-acceptor) (see Figure 1) [53]. The Lewis base (T-acceptor) can be any electron-rich entity possesing a lone pair [60,61,62,63], a π-system [55,64], an anion [44,65], etc. To explain the formation of a tetrel bond via σ-hole interactions, Politzer, Murray, and Clark suggested an interaction between a region of positive electrostatic potential as a result of diminished electron density on the tetrel atom (T-donor) and a region of negative electrostatic potential on an electron-rich atom (T-acceptor) [3,58,66,67,68]. The diminished electron density on the tetrel atom occurs as a result of electrons being mostly localized in the bonding region, which leaves a deficiency of electronic density in the outer lobe of the *p*-type valence orbital along the extension of the covalent bonding on the tetrel atom [69].

A series of experimental studies was conducted to identify and characterize tetrel bonding. The first convincing evidence of tetrel bonding was reported by Jönson and co-workers in 1975, where they observed that the carbon atoms of the carbon dioxide dimer can attractively interact with the lone pair of the oxygen of water [70] which was confirmed nine years later via microwave spectroscopic analysis by Klemperer and co-workers [71]. Recently, Guru-Row and co-workers provided experimental evidence of tetrel bonding based on an X-ray charge density analysis [43]. They revealed the existence of a bond path connecting the oxygen atom with the −CH_3_ carbon atom in R_3_N^+^−CH_3_···OH complexes [43]. Mitzel and co-workers discussed Si···N tetrel bonding in the crystalline Si(ONMe_2_)_4_ [72,73]. Evidence of tetrel bonding has also been observed by NMR spectroscopy [74]. The chemical shifts, quadrupolar couplings, and *J*-coupling are sensitive to the presence of tetrel bonding. For example, the *J*-coupling constant for (secondary) tetrel bonds has a magnitude of about 3 Hz [75].

The strength of tetrel bonding can be enhanced by cooperative effects [76,77,78] in conjunction with hydrogen bonding [79,80], halogen bonding [80], chalcogen bonding [81,82], lithium bonding [83], or with other tetrel bonding [84]. Cooperative effects in tetrel bonding [85,86,87] play an important role in crystal materials, chemical reactions, and biological systems [78,88,89]. Thus, the understanding of the strength and the nature of tetrel bonding is the key to understanding its properties. The molecular electrostatic potential ($V_{s}$) and its maximum value ($V_{s,max}$) are commonly used measures to quantify the strength of the σ-hole interaction [58,90,91]. A limited correlation between the interaction energies and the value of the $V_{s,max}$ has been reported by several authors [27,28,61,66,92]. However, very recently, Scheiner and co-workers pointed out that the maximum magnitude of the molecular electrostatic potential is not an ideal bond strength indicator [93,94]. Therefore, there is an urgent need for a qualified intrinsic bond strength descriptor, which we address in the present work.

One of the most common measures for quantifying the chemical bond strength is the bond dissociation energy (BDE) or the bond dissociation enthalpy (BDH). It has been shown that the BDE or BDH has the limitation of describing the intrinsic strength of a bond [95,96,97,98] because it includes the geometry relaxation of the fragments as well as the reorganization of the electron density. The intrinsic bond strength based on the local mode force constants $k^{a}$ measures the bond strength with only infinitesimal changes in the electronic structure of the molecule, thus excluding misleading additional contributions from the relaxation of the fragments. Many examples show that a chemical bond may have a large value of $k^{a}$ but a low BDE, vice versa [40,96,97].

**Figure 2:**
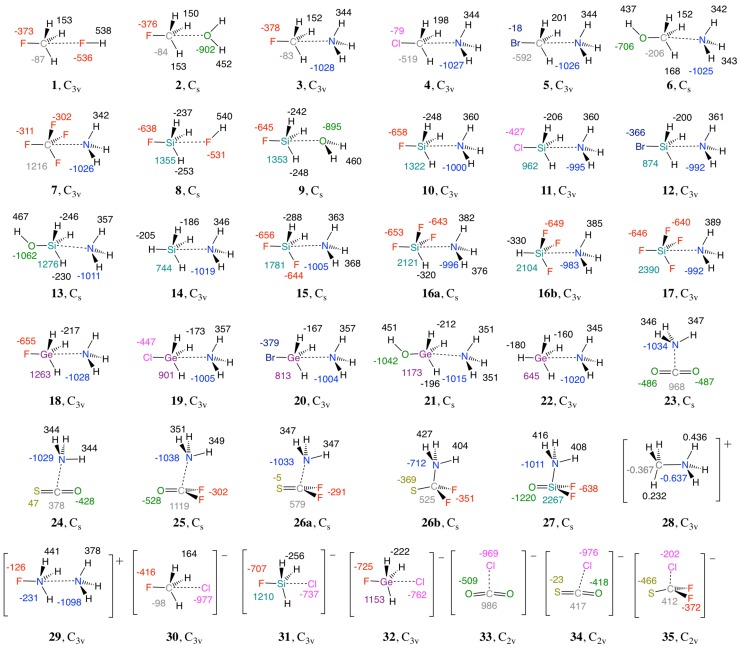
Schematic representation of complexes **1-35** with atomic charges (in me) from the natural population analysis calculated at the CCSD(T)/aug-cc-pVTZ level of theory. Colors are used to correlate charges to specific atoms.

Vibrational spectroscopy is an important tool that has been used to identify and characterize small-to-medium-sized molecules [99,100,101]. However, normal vibrational modes are of limited use as bond strength measure due to mode–mode coupling. A major breakthrough was achieved by the work of Konkoli and Cremer where the use of vibrational spectroscopy as an intrinsic bond strength measure via local vibrational modes was refined [102,103]. The intrinsic strength of chemical bonds is probed using the associated local stretching force constants $k^{a}$ [104,105,106]. The local stretching force constants $k^{a}$ have been successfully used to determine the intrinsic bond strength of covalent bonds such as CC bonds [105,107,108,109], NN bonds [110], NF bonds [98], CO bonds [111], and CX (X = F, Cl, Br, I) bonds [112,113,114,115] as well as weak chemical interactions such as hydrogen bonding [18,19,20,21,22], halogen bonding [30,116,117,118], pnicogen bonding [40,41,42], chalcogen bonding [96,97], and recently, BH···*π* interactions [119,120]. In this study, we investigate the strength and the nature of the tetrel bonds for a representative set of 35 complexes (see Figure 2) and also compare tetrel bonding with halogen and chalcogen bonding.

The main objectives of the present work are (i) to quantify the impact of changing the tetrel atom, its substituents, and the tetrel acceptor on the tetrel bond strength; (ii) to better understand the interplay between various electronic effects such as electrostatics, covalent contributions to tetrel bonding, exchange-repulsion between the tetrel acceptor and the peripheral ligands (R, R’, and R’’) of the tetrel-donor, etc.; (iii) to compare the strength of tetrel bonds with halogen (XBs), chalcogen (ChB), and pnicogen bonds (PnB); and (iv) to develop an effective strategy to tune the strength of the tetrel bond.

## 2. Computational Methods

**Figure 3:**
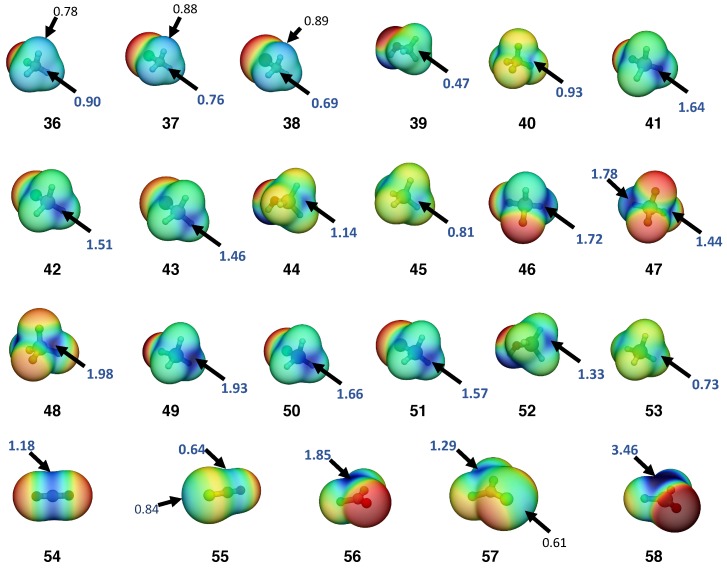
Molecular electrostatic potential of neutral tetrel-donors mapped onto the 0.001 a.u electron density surface. Blue and red correspond, respectively, to the positive and negative potential. The extreme values are ±1.9 eV. The $V_{s_{max}}$ at the tetrel σ or π-hole are given in bold blue, while the $V_{s_{max}}$ at the H (**36**, **37**, **38**) and at the chalcogen atoms (**55**, **57**) are shown in black. Calculated at the CCSD(T)/aug-cc-pVTZ level of theory.

To evaluate the key factors that influence the strength of the tetrel bonds, geometry optimizations and normal vibrational modes of complexes **1**–**35** (see Figure 2), monomers **36–60** (see Table 2, Figure 3, and Supporting Information Figure S1), and reference molecules **R1**–**R2** were calculated using the coupled cluster theory with singles, doubles, and perturbative triples (CCSD(T)) [121,122] in combination with the Dunning’s aug-cc-pVTZ basis set [123,124,125] which contains diffuse basis functions for describing the charge distribution of hetero-atoms, anions, and also, the dispersion interactions in tetrel bonds. All CCSD(T) calculations were carried out using a convergence criterion of $10^{-7}$ Hartree bohr^−1^ for geometry and a threshold of $10^{-9}$ for self-consistent field and CC-amplitudes.

Normal vibrational modes were converted into local vibrational modes using the Konkoli–Cremer method [102,103,104,107]. The electronic and mass coupling between normal vibrational modes were eliminated using the mass-decoupled analogue of the Wilson equation [107,126]. The resulting local vibrational modes, which were free from any mode-mode coupling, were associated with a given internal coordinate $q_{n}$ (bond length, bond angle, dihedral angle, etc.), which could be connected to normal vibrational modes in an one-to-one relationship via the Adiabatic Connection Scheme (ACS) [104,108]. The local force constant $k^{a}$, obtained from the corresponding local vibrational mode, was used to measure the intrinsic bond strength of the tetrel bonds.

For simplification, the local force constant $k^{a}$ was converted to the bond strength order (BSO *n*) using a power relationship of the generalized Badger rule [127]:

(1) $$n=a{\left(k^{a}\right)}^{b}.$$

The constants *a* = 0.418 and *b* = 0.564 were obtained by two references of well-defined bond order, namely the FF bond in F_2_ (*n* = 1.0) and the three-center-four-electron bond in F_3_^−^ (*n* = 0.5), as previously done in a study of halogen bonds [30] and other noncovalent interactions [117].

The binding energy was separated into two contributions:

(2) $$\Delta E=E_{int}+E_{def}.$$

$E_{int}$ is the interaction energy for the frozen geometry of the monomers, and the deformation energy ($E_{def}$) is the energetic difference between the monomers’ frozen geometry and their minimum energy geometry. The counterpoise correction (CP) [128] is usually used to eliminate the basis set superposition error (BSSE) present in $E_{int}$. However, BSSE often compensates for the error caused by an incomplete basis set; consequently, uncorrected $E_{int}$ values can be closer to the complete basis set limit (CBS) [117,129]. To test if this was the case, we compared CP-corrected and uncorrected CCSD(T)/aug-cc-pVTZ results to the ones obtained using the more saturated pentuple zeta basis set aug-cc-pV5Z [123] (see Supporting Information Table S1). The latter results were obtained by employing the domain-based local pair natural orbital (DLPNO) [130,131] approximation to CCSD(T) using a tight convergence criteria to ensure that the errors caused by the DLPNO approximation were negligible. It turned out that the uncorrected CCSD(T)/aug-cc-pVTZ values of $E_{int}$ were, on average, closer to the CP-corrected and uncorrected DLPNO-CCSD(T)/aug-cc-pV5Z results than the CP-corrected CCSD(T)/aug-cc-pVTZ values (see Supporting Information Tables S1 and S2). Therefore, in the next sections, only ΔE values without counterpoise correction are discussed (see Table 1).

CCSD(T) calculations were performed using the CFOUR program [132,133], whereas DLPNO- CCSD(T) calculations were done in ORCA 4.0 [134]. Analytical vibrational frequencies were used to verify that each equilibrium geometry obtained by CCSD(T) corresponded to a geometry minimum. The charge distribution was calculated with the natural population analysis (NPA) within the Natural Bond Orbital (NBO) scheme [135,136] using the NBO6 program [137,138]. The electron density ρ(r) and the energy density H(r) at the T···A (T: tetrel atom and A: tetrel-acceptor) electron density critical point (r) were calculated using the AIMALL program [139]. The molecular electrostatic potentials of T-donors and T-acceptors mapped onto the 0.001 e/bohr^3^ electron density surface were calculated using the Multiwfn3.5 [140] program. Noncovalent interaction (NCI) plots were calculated using the NCIplot program [141]. NBO charges, as well as, H(r), ρ(r) and V(r) were derived from CCSD(T) response densities obtained from CFOUR calculations with the help of MOLBO and Molden2AIM scripts [142]. Local mode force constants and frequencies were calculated using the COLOGNE18 program package [143].

## 3. Results and Discussion

**Figure 4:**
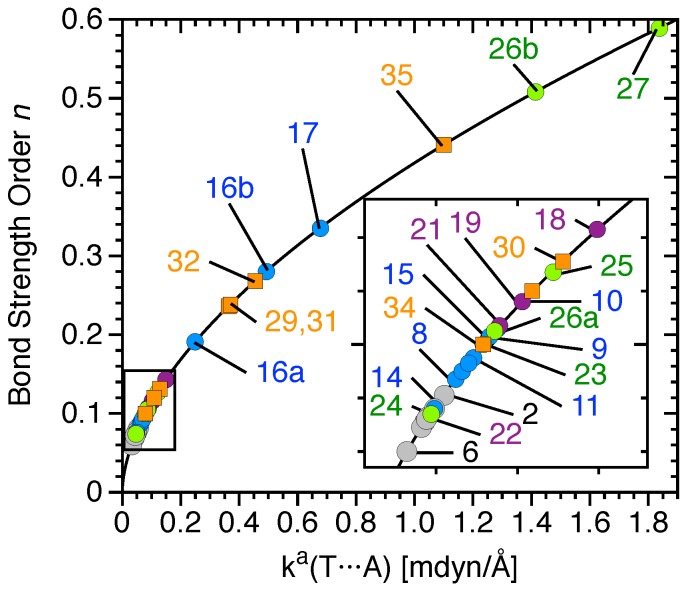
Power relationship between the relative bond strength order (BSO) *n* and the local stretching force constants $k^{a}$ of the TA interaction of complexes **1–35**. C donors are gray, Si donors are blue, Ge donors are purple, double bonded donors are green, and charge-assisted TBs are orange. Complex **28** is not shown. Calculated at the CCSD(T)/aug-cc-pVTZ level of theory.

**Figure 5:**
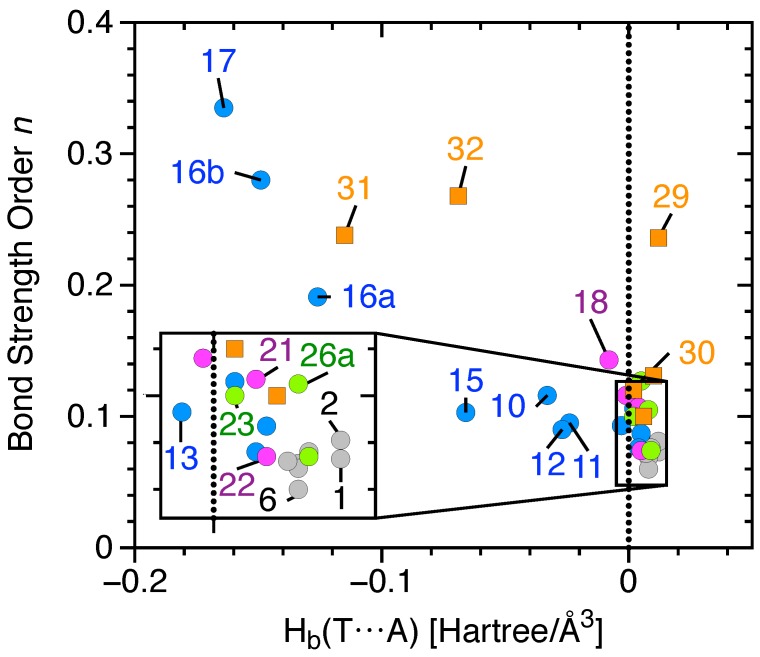
Comparison of the relative bond strength order (BSO) *n* and the energy density at the bond critical point $H_{b}$ of the tetrel bond of complexes. C tetrel bonds are gray, Si tetrel bonds are blue, Ge tetrel bonds are purple, double bonded tetrel bonds are green, and anionic tetrel bonds are orange. Complexes **26b–28** and **35** are not shown. Calculated at the CCSD(T)/aug-cc-pVTZ level of theory.

Table 1 summarizes the complex binding energy ΔE, the counterpoise corrected binding energy Δ$E_{cp}$, the monomers’ deformation energy $E_{def}$, the distance r(TA) between the tetrel-donor atom (T = C, Si or Ge) and an heteroatom (A = F, O, N or Cl^−^) of the tetrel-acceptor (T-acceptor), also called the tetrel bond (TB) distance, the distance r(XT) between the T donor atom and the donor group or atom (X = H, F, Cl, Br, OH, =O or =S), the intermonomer charge transfer (CT) obtained from the natural population analysis (NPA), the electron density $\rho_{b}$ and the energy density $H_{b}$ at the density critical point associated with TB, the local stretching force constant of TA ($k^{a}$(TA)) and XT ($k^{a}$(XT)) and the BSO *n* of the TA and XT. The calculated NBO atomic charges are given in Figure 2. T donor properties such as the maximum electrostatic potential at the σ-hole region of the tetrel atom ($V_{s_{max}}$), the total dipole moment, and the isotropic polarizability are listed in Table 2. The BSO *n* values of all TB are given as functions of their local stretching force constant $k^{a}$(T···A) in Figure 4. Similar to previous studies [30,34,40,116,117,118,144], we determined the covalent character of the TB by utilizing the energy density $H_{b}$ at the density critical point of the TB (Figure 5); electrostatic interactions were characterized by having positive $H_{b}$ values, whereas, according to the Cremer–Kraka criterion, [145,146], covalent interactions have negative $H_{b}$ values, indicating that the accumulated electron density at the interactions region stabilizes the complex. Although the relationship between BSO *n* and $H_{b}$ is scattered, the TB strength tends to increase with the increasing covalent character of the interaction, especially among neutral complexes.

### 3.1. Tetrel Bonds (TB) in Neutral Complexes

**TBs involving C donors:** Tetrel bonds (TBs) involving a neutral sp^3^ hybridized carbon as a tetrel donor and neutral tetrel acceptors (complexes **1–7**) have weak interactions (BSO *n*≤ 0.081; ΔE≥−2.25 kcal/mol). The energy density at the TB density critical point is destabilizing ($H_{b}$≥ 0.007 Hartree/Å^3^), indicating that these TBs are electrostatic in nature. TB with C donors that have peripheral H ligands (**1–6**) are not only stabilized by a lone pair of the T acceptor lp(A)-σ-hole electrostatic attraction but also by the electrostatic attraction between the positive charge at the hydrogen atoms (Hs) and the negative charge at lp(A) (see the NPA atomic charges in Figure 2 and the monomers’ electrostatic potential in Table 2 and Figure 3). The presence of the later interactions were also verified by noncovalent interaction plots (NCIs plots), which showed a weak (attractive) electrostatic interaction (see Supporting Information Figure S2). Complex **7** (CF_4_···NH_3_) shows that an attractive interaction can be formed even in the absence of positively charged Hs on the T-donor. However, in **7**, there is no electron density path connecting the N of the T acceptor to the C of the T donor indicating the formation of a very weak dispersive interaction, as pointed by Grabowski [147]. The calculated spectra of **7** clearly shows an intermonomer stretching vibration of A_1_ symmetry at 73 cm^−1^. Decomposition of the normal vibrational mode into the local vibrational mode shows that the CN local stretching mode contributes solely to this normal vibrational mode, confirming the existence of tetrel bonding in **7**. As revealed by the NCI plot analysis, the peripheral H ligands of the T acceptor have an additional weak (attractive) electrostatic interaction with the peripheral F ligands of the T donor, which provides additional stabilization to the complex (see Supporting Information Figure S2).

The TB strength in the series of C donors and neutral T acceptors (**1–7**) shows only a small variation (0.021 for *n* and 0.87 kcal/mol for ΔE) which is affected by several factors such as the positive charge at the Hs (**5**>**4**>**3**≈**6**), the negative electrostatic potential at lp(A) of the T-acceptor (NH_3_<OH_2_< FH), and the intermonomer distance (e.g., 3.035 Å (**2**) compared to 3.218 (**3**)). It is noteworthy that for X−CH_3_···NH_3_ complexes, the TB strength weakens in the order of **3** (X = F) >**4** (X = Cl) >**5** (X = Br) >**6** (X = OH) as the magnitude of the σ-hole decreases (**36** (F−CH_3_)) >**37** (Cl−CH_3_) >**38** (Br−CH_3_) > **39** (OH−CH_3_) (see Figure 3).

**TB involving Si donors:** The strength of complexes **8–14** can be understood mostly on the basis of the extreme values of the electrostatic potential of the monomers. First, by varying the T-acceptor in FSiH_3_···A, where A = FH (**8**), OH_2_ (**9**), NH_3_ (**10**), the TB strength trends follows the increase in magnitude of the negative potential at the lp(A) of the Lewis base. It is noticeable that $V_{s,min}$(A) and $V_{s,max}$(T) in **9** are not aligned as in **2**, indicating that even in this case, the stabilization brought by electron delocalization involving the highest occupied orbital HOMO of H_2_O (the lp(O) orbital of B_1_ symmetry, see Supporting Information Figure S3) and the lowest unoccupied orbital of FSiH_3_ ($\sigma^{⋆}$(SiF) orbital) can influence the geometry of the complex (see Supporting Information Figure S4). Second, by varying the T donors in the XSiH_3_···NH_3_ complexes, the TB strength decreases in the order **10** (X = F) > **11** (X = Cl) > **12** (X = Br) ≈**13** (X = OH) > **14** (X = H) as the Vs at the σ-hole decreases (**41** (F−SiH_3_) > **42** (Cl−SiH_3_) > **43** (Br−SiH_3_) > **44** (HO−SiH_3_) > **45** (SiH_4_)). The only exception is **44**, which has a more positive NBO charge at Si (1277 me compared to 852 me in **43**) but a less positive $V_{s,max}$ (1.14 (**44**) compared to 1.46 (**43**), see Table 2 and Supporting Information Figure S1). This could be caused by the stronger σ(O-Si) orbital contraction, whereby the Si atom in HOSiH_3_ is more electron-deficient than the Si atom in BrSiH_3_, but due to the higher electronegativity of O, the σ(O-Si) is more compact than σ(Br-Si), resulting in a better shielded Si nucleus, reflected in the less positive potential at the σ-hole region (given by $V_{s,max}$). Substituting F in FSiH_3_ would lead to an even stronger σ(F-Si) orbital contraction, but this effect would be overcome by the higher electron deficiency of Si.

**Stepwise fluorination of SiH_4_:** The successive fluorination of SiH_4_ (complexes **10** and **15–17**) impacts both the strength and the nature of the TB. Substituting the H collinear to the TB in complex **14** results in complex **10**, which has a stronger TB interaction due to the higher $V_{s}$ at the σ-hole region and is due to the partial covalent character of the interaction ($H_{b}$ = −0.033 Hartree/Å for complex **10**), which can be understood on the basis of molecular orbital interactions, as the electron delocalization from the highest occupied molecular orbital (HOMO) of the NH_3_ (lone pair orbital of N, lp(N)) into the lowest unoccupied molecular orbital (LUMO) of FSiH_3_ (a $\sigma^{⋆}$(FSi) orbital see Supporting Information Figure S5).

A second fluorine substituent (complex **15**) shows three different electronic effects: (i) the second fluorine withdraws the electron density from Si, decreasing its covalent radius and thus increasing the $V_{s}$ at the σ-hole region, resulting in a stronger σ-hole-lp(N) electrostatic attraction; (ii) the lp(N)$\to \sigma^{⋆}$(SiF) electron delocalization is not restricted to the $\sigma^{⋆}$ orbital of the Si–F bond that is collinear to the lp(A) but can also take place to the $\sigma^{⋆}$ orbital of the second Si–F bond (see Table 3); (iii) the orbital effect of the exchange-repulsion between the lone pair orbitals of the peripheral fluorine lp(F) and lp(N) orbital. Effects (i) and (ii) are responsible for the 0.123 Å shorter TB in **15** compared to **10**. However, due to effect (iii), complexes **10** and **15** have similar TB strengths (*n* = 0.116 for **10**, 0.103 for **15**; ΔE = 6.8 kcal/mol for **10**, and 7.0 kcal/mol for **15**).

The addition of a third fluorine substituent (complexes **16a** and **16b**) leads to shorter and stronger TBs (BSO *n* = 0.191 (**16a**) and 0.280 (**16b**) compared to 0.103 (**15**)). However, this great increase in the TB strength (especially for **16b**) is not reflected by the binding energies of **16a** and **16b** (ΔE = −7.7 kcal/mol for **16a** and −6.3 kcal/mol for **16b**). The reason for the unexpectedly low ΔE values of **16a** and **16b** is due to the energetic cost associated with the geometric deformation of the monomers upon complexation ($E_{def}$ = 11.8 kcal/mol for **16a**, 21.2 kcal/mol for **16b**). Monomer deformation is mostly caused by the lp(F)–lp(N) exchange-repulsion (effect (iii)), which pushes the peripheral ligands towards the bond collinear to the TB. For example, there is a decrease of 12.3° in the H–Si–F bond angle of HSiF_3_ upon the formation of **16b**. Monomer deformation and the steric effect on TB complexes were also topics of a recent study carried by Scheiner [148,149]. It is noteworthy that the strongest TB between SiF_3_H and NH_3_ is collinear to the $V_{s}$ at the σ-hole of the Si–H bond (complex **16b**), instead of the most positive potential at the σ-hole of the Si–F bond as one would expect from the σ-hole model or from steric considerations. The stronger and more covalent bond in **16b** is due to the higher stabilization energies brought by electron delocalization from lp(N) into $\sigma^{⋆}$(FSi) and into the $\sigma^{⋆}$(HSi) unoccupied orbital, as shown in Table 3. Even if the TB of **16b** is elongated to match the TB distance of **16a**, the NBO second-order delocalization energies of **16b** are still higher than those of **16a** (see Table 3).

The addition of a fourth fluorine (complex **17**) makes the TB even stronger (*n* = 0.335 for **17**), compared to 0.280 for **16**), which is a consequence of the more positive $V_{s}$ and of the higher electron delocalization that occurs from lp(N) to $\sigma^{⋆}$(FSi) compared to $\sigma^{⋆}$(H-Si) (see Table 3 and Supporting Information Figure S5). The substitution of the H collinear to the TB in **16b** by a fluorine does not increase the steric repulsion between the monomer ($E_{def}$ of **17** is almost the same of **16b**), and as a result, ΔE is 5.1 kcal/mol more stable.

**TB involving Ge donors:** The germanium electron density is more easily polarized by an electronegative substituent than silicon. As a result, $V_{s}$ at the σ-hole of Ge-donors (**49–52**) are higher than Si donors (see Table 2), the only exception being GeH_4_ (**53**), which is a consequence of the higher electronegativity of Ge (χ(Si): 1.74 compared to χ(Ge): 2.02). Due to the stronger lp(N)-σ-hole electrostatic attraction, mono-substituted Ge-donors (**18**, **19**, **20**, **21**) form stronger TBs than mono-substituted Si donors (**10**, **11**, **12**, **13**) when paired with the NH_3_ T acceptor. Conversely, the covalent component of this interaction is slightly reduced because of the more diffuse nature of Ge orbitals (see CT and $H_{b}$ values on Table 1). Similar to C donors and Si donors, for X−GeH_3_···NH_3_ complexes, the TB strength decreases in the order of **18** (X = F) >**19** (X = Cl) >**20** (X = Br) >**21** (X = OH), as the magnitude of the σ-hole decreases (**49** (F−GeH_3_)) >**50** (Cl−GeH_3_) >**51** (Br−GeH_3_) > **52** (OH−GeH_3_) (see Table 2 and Figure 3).

**TB in double bonded C and Si donors:** When a carbon atom forms a π-bond, density is moved from a p-orbital of the carbon into the π-bond, resulting in a depletion of electron density at the C and the formation of a region of positive potential called a π-hole [150,151]. As noted previously by various authors [152,153,154], the lp(A)-π-hole electrostatic attraction is an important component of the TB involving double bonded C, Si donors. Another important characteristic of these T-donors is the existence of a low lying empty $\pi^{⋆}$(XT) orbital which is capable of accepting electron density from the lp(A) orbital of the T-acceptor. In order to evaluate strategies to strengthen TB involving double bonded C and Si donors, complexes **23–27** were investigated. Due to the $D_{\infty h}$ and $C_{\infty v}$ symmetry of CO_2_ (**54**) and SCO (**55**), respectively, the π-bond density of these monomers has a central constriction with a negative $V_{s}$, leaving a belt-shaped π-hole around the C atom (see Figure 3). In SCO (**55**), a chalcogen bond is also possible due to the formation of a positive $V_{s}$ at the σ-hole of sulfur. The TBs between CO_2_ (**23**) and SCO (**24**) T donors and the prototypical T acceptor NH_3_ are weak (*n* = 0.100 (**23**), 0.074 (**24**)) and electrostatic in nature ($H_{b}>$ 0.002 Hartree/Å).

**Figure 6:**
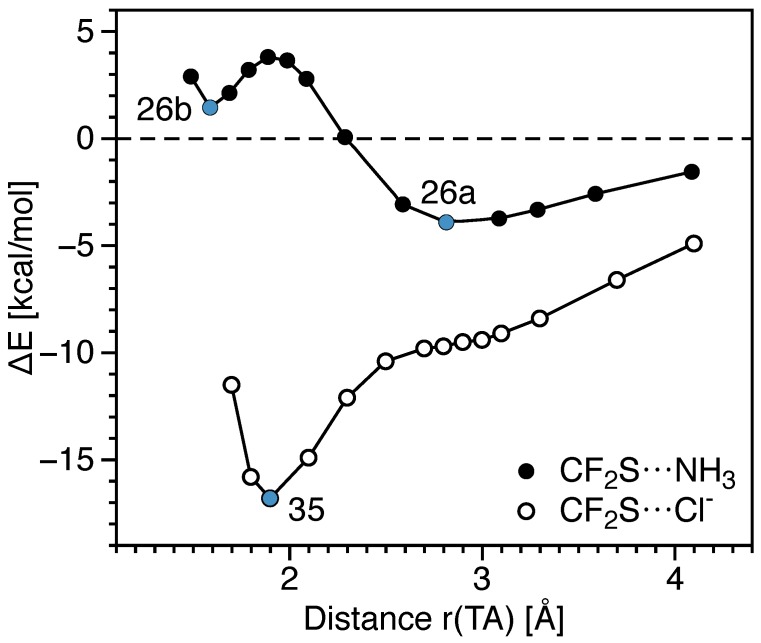
Relationship between the binding energy and interactomic distance computed at the CCSD(T)/aug-cc-pVTZ level of theory. All geometric parameters were optimized at each point of the curves for fixed r(TA) values. The blue dots represent the binding energy at the minima of complex **26** and the minimum of **35**; the black lines connecting points were used to improve interpretation.

Substituting a CO double bond by two CF single bonds in **23** and **24** results in complexes CF_2_O···NH_3_ (**25**) and CF_2_S···NH_3_ (**26**), respectively. The T-donors of these complexes are characterized by having a higher $V_{s}$ at the π-hole ($V_{s}$ = 1.85 eV (**56**), 1.29 eV (**57**) compared to 1.18 eV (**54**) and 0.64 eV (**55**)), resulting in stronger TBs (*n* = 0.127 (**25**), 0.105 (**26a**) compared to 0.100 (**23**) and 0.074 (**24**)). The atypically strong (*n* = 0.508), highly covalent ($H_{b}$ = −1.339 Hartree/Å ${}^{3}$) and short interactions (r(TA) = 1.7 Å) found in complex **26b** are formed at the expense of breaking the CS π-bond (*n*(C=S) decreases from 1.214 in **57** to 0.891 in **26b**). The energetic cost involved in the deformation of the monomers of **26b** is 1.45 kcal/mol higher than the stabilization brought by complexation ($E_{int}$); hence, the **26b** is less stable than the separated monomers. A small energetic barrier in the dissociative direction separates **26b** from the electrostatic TB complex **26a** (see Figure 6). An even stronger (*n* = 0.589) but less covalent interaction ($H_{b}$ = −0.224 Hartree/Å ${}^{3}$) is formed between SiF_2_O and NH_3_ (complex **27**). In this complex, the SiO double bond is kept almost unaltered (*n* = 1.465 (**58**); 1.425 (**27**)); consequently, the deformation energy is relatively low compared to the stabilization energy brought by the complexation, resulting in a binding energy of −44.14 kcal/mol.

### 3.2. Charge-Assisted Tetrel Bonds

**Charge-assisted interactions:** Similar to other NCIs [30,34,116,118,155], TBs can be strengthened by having a positively-charged T donor or a negatively-charged T acceptor **30**–**35**. The simplest positively charged C donor is CH_3_^+^ (isoelectronic to BH_3_). However, due to the availability of an empty *p*-orbital to coordinate with the lone pairs of the NH_3_, the C–N bond in **28** clearly differs from the tetrel bonds. This covalent bond in [CH_3_−NH_3_]^+^ (**28**) is much stronger (*n* = 0.882; ΔE = −110.26 kcal/mol) and covalent ($H_{b}$ = −1.952 Hartree/Å ${}^{3}$) compared to a TB. The existence of a covalent bond in complex **28** is also confirmed by the NCI plot, showing that there is no noncovalent interaction (see Supporting Information Figure S2). On the other hand, the cationic pnicogen donor FNH_3_^+^, isoelectronic to FCH_3_, forms a noncovalent interaction with the NH_3_ (complex **29**) which closely resembles the ones formed by neutral C donors (**1–7**) characterized by an electrostatic nature ($H_{b}$) and an interaction collinear to the X–T bond. The only difference is the higher $V_{s}$ at the σ-hole ($V_{s}$ = 8.58 eV), which results in a stronger electrostatic interaction (*n* = 0.236; ΔE = −23.14 kcal/mol).

An increase in the TB strength of as much as 105% (**31**) occurs for complexes involving a neutral T donor and a chloride anion as a T acceptor. This increase does not affect TB strength trends, such as FGeH_3_ >FSiH_3_>FCH_3_, and CO_2_>SCO, nor the trends in the covalent character of the TB in these series. Conversely, complex CF_2_S forms a weaker bond with Cl^−^ compared to NH_3_ (*n* = 0.441 (**35**) 0.508 (**26b**)), but has a highly negative binding energy (ΔE = −16.81 kcal/mol of **35** compared to the 1.45 kcal/mol of **26b**). The inverse relationships between the bond strength and interaction energies ($E_{int}$) or binding energies (ΔE) between these complexes indicate that the local C–N or C–Cl stretching is not the determining factor for the complex stabilization. Other components of the interaction which do not contribute to the strength of T-A local stretching also stabilize the complex. Figure 6 shows the dissociation curves for the CF_2_S···NH_3_ (**26**) and CF_2_S···Cl^−^ (**35**). Only **26** has a minimum geometry with an electrostatic TB (**26a**), whereas the dissociation curve for **35** has a flat region around r(TA) = 2.9Å separating the electrostatic interaction found for long TBs from the strong covalent interactions in **35**. The barrier energy from **26a** to **26b** is about 3 kcal/mol.

### 3.3. Tetrel Bonds vs. Other Noncovalent Interactions

We also compared TB with other noncovalent interactions, such as halogen (XB), chalcogen (ChB), and pnicogen (PnB), in mono-fluorinated systems involving the third period series FCl, FSH, FPH_2_ with a medium (OH_2_) and a strong (NH_3_) Lewis base (see Table 4). It was clearly shown that the TB formed by FSiH_3_ tend to be weaker than the other noncovalent interactions, the only exception being FSiH_3_···Cl^−^ (**31**) (*n* = 0.238) which is slightly stronger than FPH_2_···Cl^−^ (*n* = 0.214) but weaker than FSH···Cl^−^ (*n* = 0.264) and FCl···Cl^−^ (*n* = 0.382). Increasing the polarizability of the T donor moving FSiH_3_ to FGeH_3_ does increase the strength of the tetrel bond enough to compete with the halogen bonds formed by FCl. A better strategy for obtaining TBs that are strong enough to compete with halogen bonds and other noncovalent interactions is the substitution of peripheral Hs in FSiH_3_ by fluorine atoms. SiF_4_···NH_3_ (**17**), for example, has a BSO of *n* = 0.335 compared to *n* = 0.216 for the FCl···NH_3_. A clear advantage of tetrel bonding is that even in the absence of a strong polarizing group collinear to the TB, such as in HSiF_3_···NH_3_, the TB is still stronger than other noncovalent interactions (*n* = 0.280 for **16b**). This TB feature should be extensively explored to tune the strength of TB involving not only Si donors but also the heavier and more polarizable Ge and Sn donors.

## 4. Conclusions

In the present work, we investigated a set of 35 representative tetrel complexes (ΔE = −1.4 to −26 kcal/mol) with the objective of finding the factors that influence the strengths of neutral and charged tetrel bonds involving C donors, Si donors, Ge donors, and double bonded C or Si donors. The strength of a tetrel bond is affected by the complex interplay of several factors, such as the magnitude of the σ-hole in the tetrel atom, the negative electrostatic potential at the lp(A) of the T acceptors, the positive charge at the peripheral hydrogen (Hs) of the T donors, exchange-repulsion between the lone pair orbitals of the peripheral atoms of the T donor, and the covalent character which can be rationalized on the basis of electron delocalization from the highest occupied molecular orbital (HOMO) of the T acceptor into the lowest unoccupied orbitals (LUMOs) of the T donor, which is not limited to $\sigma^{⋆}$(X−T) orbital but can also involve the peripheral substituents (orbital of $\sigma^{⋆}$(R−Si) character), allowing the formation of strong tetrel bonds, even in the absence of an electronegative X substituent collinear to the TB. This clearly shows that focusing on just one or two of these factors, in particular, the σ-hole description, can only lead to an incomplete picture [93,94,156,157]. In this work, we derived, for the first time, the intrinsic bond strength of tetrel bonds from calculated vibrational spectroscopy data, which, combined with NBO charges, charge transfer, dipole moments, electrostatic potentials, electron and energy density distributions, difference density distributions, and noncovalent interaction plots calculated at the CCSD(T)/aug-cc-pVTZ level of theory, led to a complete insight into how different electronic effects influence the intrinsic strength of the tetrel bonding.

- Tetrel bonding becomes stronger as the atomic mass of the tetrel center increases as a consequence of increasing the polarizability.
- For X−TH_3_···NH_3_ complexes, the tetrel bond strength weakens in the order (X = F) > (X = Cl) > (X = Br) ≥ (X = OH) as the magnitude of the σ-hole decreases in the order of F−TH_3_>Cl−TH_3_>Br−TH_3_≥ OH−TH_3_.
- Successive fluorination of SiH_4_ impacts both the strength and the nature of the tetrel bond. The successive fluorinations result in stronger tetrel bonding as a consequence of (i) higher $V_{s}$ at the σ-hole region; (ii) the partial covalent character of the interaction; (iii) higher electron delocalization that occurs from the highest occupied molecular orbital (HOMO) of the T acceptor to the lowest unoccupied molecular orbital (LUMO) of the T donor. In this series, the binding energy trend deviates from BSO *n* values due to the high energetic cost associated with the geometric deformation of the monomers upon complexation ($E_{def}$) which is a consequence of the exchange-repulsion between the lone pair orbitals of the peripheral atoms of the T donor.
- Tetrel bonds in double bonded C donors, e.g., CO_2_ with NH_3_, are weak and electrostatic in nature. Substituting a C=O double bond with an electron withdrawing group (F atoms) strengthens the tetrel bond.
- A positively-charged Tdonor or negatively-charged T-acceptor strengthens the tetrel bond. It creates higher $V_{s}$ at the σ-hole, resulting in a stronger electrostatic interaction.

We suggest that future materials based on strong tetrel bonding should be based on Si or heavier tetrel atoms, such as Ge and Sn, combined with peripheral fluorine ligands. Due to the larger size of Ge and Sn, the deformation energy in XGeF_3_···NH_3_ or XSnF_3_···NH_3_ should be smaller than XSiF_3_···NH_3_, making these complexes substantially more stable than XSiF_3_···NH_3_.

Although all complexes discussed in this paper represent the most stable tetrel-bonded complexes, not all of them represent the most stable structure possible (global minimum). For example, the hydrogen bonded complexes FH···CFH_3_ (**1**), OH_2_···CF_3_H (**2**), NH_3_···CF_3_H (**3**) are more stable than the tetrel-bonded complexes. However, a detailed analysis of the competition between tetrel bonds and other noncovalent interactions will be studied in the near future.

## Figures and Tables

**Table 1:** Summary of energetics, electron density, energy density, geometric, bond strength order, and vibrational spectroscopy data for complexes 1–35 *.

#	Complex (symm.)	ΔE	$\Delta E_{\mathrm{cp}}$	$E_{\mathrm{def}}$	r	r	CT	$\rho_{b}$	$H_{b}$	$k^{a}$	n	$k^{a}$	n
					TA	XT		TA	TA	TA	TA	XT	XT
**Neutral tetrel bonds involving C donors**													
**1**	FCH_3_···FH (C_3v_)	−1.50	−1.29	0.01	2.972	1.392	2	0.034	0.012	0.045	0.073	5.018	1.038
**2**	FCH_3_···OH_2_ (C_s_)	−2.10	−1.87	0.02	3.035	1.394	3	0.041	0.012	0.055	0.081	4.956	1.030
**3**	FCH_3_···NH_3_ (C_3v_)	−2.25	−2.05	0.02	3.218	1.395	5	0.040	0.009	0.049	0.076	4.912	1.025
**4**	ClCH_3_···NH_3_ (C_3v_)	−2.08	−1.88	0.02	3.289	1.798	6	0.037	0.008	0.043	0.071	2.943	0.768
**5**	BrCH_3_···NH_3_ (C_3v_)	−2.00	−1.80	0.02	3.304	1.953	6	0.037	0.008	0.041	0.069	2.515	0.703
**6**	(HO)CH_3_···NH_3_ (C_s_)	−1.38	−1.21	0.01	3.362	1.429	3	0.031	0.008	0.032	0.060	4.652	0.994
**7**	CF_4_···NH_3_ (C_3v_)	−1.62	−1.24	0.06	3.426	1.328	1	0.030 ${}^{a}$	0.007 ${}^{a}$	0.044	0.072	5.926	1.140
**Neutral tetrel bonds involving Si donors**													
**8**	FSiH_3_···FH (C_s_)	−2.28	−1.85	0.06	2.964	1.617	9	0.055	0.005	0.062	0.087	4.970	1.032
**9**	FSiH_3_···OH_2_ (C_s_)	−4.20	−3.61	0.35	2.774	1.623	25	0.092	0.002	0.088	0.106	4.762	1.007
**10**	FSiH_3_···NH_3_ (C_3v_)	−6.80	−5.94	2.11	2.523	1.637	81	0.179	−0.033	0.103	0.116	4.209	0.940
**11**	ClSiH_3_···NH_3_ (C_3v_)	−6.13	−5.41	2.02	2.580	2.117	84	0.165	−0.024	0.073	0.095	1.941	0.607
**12**	BrSiH_3_···NH_3_ (C_3v_)	−6.11	−5.35	2.23	2.566	2.290	90	0.170	−0.027	0.066	0.090	1.505	0.526
**13**	(HO)SiH_3_···NH_3_ (C_s_)	−4.13	−3.61	0.68	2.825	1.680	42	0.108	−0.003	0.070	0.093	4.065	0.921
**14**	SiH_4_···NH_3_ (C_3v_)	−2.27	−1.97	0.15	3.202	1.490	18	0.060	0.004	0.049	0.076	2.793	0.746
**15**	SiF_2_H_2_···NH_3_ (C_s_)	−6.99	−5.73	4.74	2.400	1.613	95	0.225	−0.066	0.083	0.103	4.573	0.985
**16a**	SiF_3_H···NH_3_ (C_s_)	−7.66	−5.77	11.77	2.205	1.617	139	0.320	−0.126	0.249	0.191	4.698	1.000
**16b**	HSiF_3_···NH_3_ (C_3v_)	−6.30	−4.14	21.22	2.104	1.474	172	0.390	−0.149	0.493	0.280	2.974	0.772
**17**	SiF_4_···NH_3_ (C_3v_)	−11.40	−8.86	21.15	2.072	1.609	176	0.419	−0.164	0.678	0.335	5.046	1.041
**Neutral tetrel bonds involving Ge donors**													
**18**	FGeH_3_···NH_3_ (C_3v_)	−7.77	−7.18	1.40	2.624	1.816	44	0.169	−0.008	0.149	0.143	4.125	0.929
**19**	ClGeH_3_···NH_3_ (C_3v_)	−6.22	−5.75	1.07	2.755	2.216	64	0.134	−0.001	0.103	0.116	1.921	0.604
**20**	BrGeH_3_···NH_3_ (C_3v_)	−6.01	−5.53	1.07	2.776	2.375	66	0.132	0.000	0.097	0.112	1.591	0.543
**21**	(HO)GeH_3_···NH_3_ (C_s_)	−4.58	−4.18	0.50	2.910	1.818	39	0.101	0.004	0.089	0.107	3.49	0.845
**22**	GeH_4_···NH_3_ (C_3v_)	−1.99	−1.79	0.09	3.323	1.550	15	0.052	0.005	0.047	0.074	2.580	0.713
**Neutral tetrel bonds involving double bonded C or Si donors**													
**23**	CO_2_···NH_3_ (C_s_)	−3.09	−2.84	0.11	2.922	1.167	5	0.107	0.002	0.079	0.100	15.183	1.938
**24**	SCO···NH_3_ (C_s_)	−1.97	−1.69	0.02	3.209	1.573	3	0.046	0.009	0.047	0.074	7.081	1.260
**25**	CF_2_O···NH_3_ (C_s_)	−5.55	−4.82	0.27	2.687	1.178	12	0.113	0.005	0.122	0.127	14.393	1.880
**26a**	CF_2_S···NH_3_ (C_s_)	−3.91	−3.23	0.11	2.897	1.607	9	0.078	0.008	0.086	0.105	6.397	1.190
**26b**	CF_2_S···NH_3_ (C_s_)	1.45	4.28	24.13	1.587	1.701	545	1.388	−1.339	1.414	0.508	3.828	0.891
**27**	SiF_2_O···NH_3_ (C_s_)	−44.14	−42.16	7.96	1.917	1.529	229	0.569	−0.224	1.838	0.589	8.803	1.425
**Charge-assisted interactions**													
**28**	CH_3_^+^···NH_3_ (C_3v_)	−110.25 ${}^{b}$	−109.01	24.95	1.511	1.087	329	1.517	−1.952	3.766	0.882	5.458	1.088
**29**	FNH_3_^+^···NH_3_ (C_3v_)	−23.14	−22.77	0.43	2.619	1.374	35	0.142	0.012	0.364	0.236	5.226	1.062
**30**	FCH_3_···Cl^−^ (C_3v_)	−9.77	−9.34	0.39	3.179	1.419	23	0.064	0.010	0.128	0.131	4.155	0.933
**31**	FSiH_3_···Cl^−^ (C_3v_)	−20.73	−19.49	12.03	2.504	1.703	263	0.277	−0.115	0.370	0.238	2.793	0.746
**32**	FGeH_3_···Cl^−^ (C_3v_)	−26.10	−25.09	10.71	2.566	1.892	238	0.290	−0.069	0.455	0.268	2.451	0.693
**33**	CO_2_···Cl^−^ (C_s_)	−7.45	−6.99	1.44	2.920	1.170	31	0.107	0.002	0.109	0.120	14.879	1.916
**34**	SCO···Cl^−^ (C_s_)	−5.36	−4.96	0.52	3.143	1.581	24	0.073	0.006	0.079	0.100	6.568	1.208
**35**	CF_2_S···Cl^−^ (C_s_)	−16.81	−13.83	32.63	1.898	1.725	798	1.031	−0.593	1.100	0.441	3.414	0.835

* Binding energies (ΔE), counterpoise corrected binding energies ΔE_*cp*_ and monomers’ deformation energies upon complexation (*E_def_*) in kcal/mol. XT bond distance r(XT) and tetrel bond distance r(TA) in Å. Density at the TA critical point *ρ_b_* in e/Å^3^, energy density at the TA critical point H_*b*_ in Hartree/Å^3^. Natural population analysis (NPA) charge transfer in mili-electrons (me). TA and XT local stretching force constant (k*^a^*) in mdyn/Å and bond strength order (BSO) *n* values. Computed at the CCSD(T)/aug-cc-pVTZ level of theory. ^a^ Calculated at a cage critical point (see Ref. [147]). ^b^ Covalent bond, see text.

**Table 2:** Geometry, vibrational spectroscopy data, and values of the electrostatic potential for the monomers *.

#	Monomers	$V_{s_{\max}}$(X)	r(XT)	$k^{a}$(XT)	n(XT)	Dipole	$\alpha_{\mathrm{iso}}$
**36**	F−CH_3_	0.90	1.389	5.107	1.048	1.88	2.5
**37**	Cl−CH_3_	0.76	1.792	3.068	0.786	1.92	4.3
**38**	Br−CH_3_	0.69	1.948	2.616	0.718	1.86	5.4
**39**	HO−CH_3_	0.47	1.426	4.749	1.006		3.1
**40**	F−CF_3_	0.93	1.321	6.204	1.170	0.00	2.8
**41**	F−SiH_3_	1.64	1.613	5.120	1.049	1.38	4.1
**42**	Cl−SiH_3_	1.51	2.072	2.799	0.746	1.41	6.2
**43**	Br−SiH_3_	1.46	2.238	2.321	0.672	1.38	7.4
**44**	HO−SiH_3_	1.14	1.664	4.517	0.978		4.9
**45**	H−SiH_3_	0.81	1.483	2.903	0.762	0.00	4.6
**46**	F−SiH_2_F	1.72	1.597	5.497	1.092		3.5
**47a**	F−SiF_2_H	1.78	1.583	5.884	1.135		3.8
**47b**	H−SiF_3_	1.44	1.458	3.273	0.815	1.43	3.8
**48**	F−SiF_3_	1.98	1.571	6.281	1.178	0.00	3.3
**49**	F−GeH_3_	1.93	1.793	4.951	1.030	2.25	4.7
**50**	Cl−GeH_3_	1.66	2.175	2.491	0.699	2.04	6.9
**51**	Br−GeH_3_	1.57	2.330	2.091	0.633	1.93	8.1
**52**	HO−GeH_3_	1.33	1.802	3.872	0.896		5.5
**53**	H−GeH_3_	0.73	1.542	2.693	0.730	0.00	5.2
**54**	O=CO	1.18	1.167	15.613	1.969	0.00	2.6
**55**	S=CO	0.64	1.575	7.227	1.275	0.68	5.2
**56**	O=CF_2_	1.85	1.177	14.680	1.902	1.00	2.8
**57**	S=CF_2_	1.29	1.603	6.626	1.214	0.16	5.2
**58**	O=SiF_2_	3.46	1.517	9.243	1.465	2.31	4.0
**59**	CH_3_^+^	10.01				0.00	1.3
**60**	F−NH_3_^+^	8.58	1.368	5.642	1.109	4.78	1.7

* Maximum electrostatic potential at the σ-hole of X ($V_{s_{max}}$(X)) in eV. XT bond distance r(XT) in Å, XT local stretching force $k^{a}\left(XT\right)$ in mdyn/Å , XT bond strength order n(XT). Dipole moment in Debye and static isotropic polarizability in Å${}^{3}$. All values were calculated with CCSD(T)/aug-cc-pVTZ.

**Table 3:** Naural Bond Orbital (NBO) electron delocalization energies involving the lone pair of NH_3_ *.

#	Complex	$\sigma^{⋆}$(X-Si)	$\sigma^{⋆}$(Si-R)	$\sigma^{⋆}$(Si-R’)	$\sigma^{⋆}$(Si-R”)
**10**	FSiH_3_···NH_3_	15.7	2.4	2.4	2.4
**15**	SiF_2_H_2_···NH_3_	12.7	6.2	3.1	3.1
**16a**	SiF_3_H···NH_3_	16.3	11.9	11.9	7.5
**16b**	SiF_3_H···NH_3_	11.4	23.5	23.5	23.5
**16b** ${}^{a}$	SiHF_3_···NH_3_	7.9	16.8	16.8	16.8
**17**	SiF_4_···NH_3_	20.7	19.5	19.5	19.5

* NBO electron delocalization energies from the second-order perturbation analysis referent to the interaction involving the lp(N) orbital of NH_3_ and the $\sigma^{⋆}$(X-Si) (collinear to the TB), the $\sigma^{⋆}$(Si-R), $\sigma^{⋆}$(Si-R’) and the $\sigma^{⋆}$(Si-R”) (peripheral to the TB, see Figure 1) of selected tetrel donors (see Supporting Information Figure S5). Values are in kcal/mol. Calculated with ωB97XD/aug-cc-pVTZ. ^a^ Complex **16b** with an elongated tetrel bond (TB) to match the TB distance of **16a**.

**Table 4:** Summary of energetics, geometric and vibrational spectroscopy data for other types of interactions *.

Complex	ΔE	$\Delta E_{\mathrm{cp}}$	r	CT	ρ	$H_{b}$	$k^{a}$	n	$k^{a}$	n	
			TA		TA	TA	TA	TA	XT	XT	
F_2_···OH_2_ (C_s_)	−1.42	−1.15	2.662	0.005	0.066	0.022	0.057	0.083	4.488	0.974	
Cl_2_···OH_2_ (C_s_)	−2.98	−2.62	2.808	0.015	0.098	0.018	0.097	0.112	2.896	0.761	
FCl···OH_2_ (C_s_)	−5.22	−4.75	2.566	0.032	0.163	0.016	0.170	0.154	3.967	0.909	
FSH···OH_2_ (C_s_)	−5.69	−5.15	2.659	0.028	0.138	0.010	0.152	0.144	4.011	0.914	
FPH_2_···OH_2_ (C_s_)	−4.63	−4.02	2.780	0.021	0.107	0.006	0.118	0.125	4.198	0.938	
F_2_···NH_3_ (C_3v_)	−2.00	−1.69	2.615	0.017	0.097	0.027	0.062	0.087	3.821	0.890	
Cl_2_···NH_3_ (C_3v_)	−4.92	−4.43	2.664	0.055	0.172	0.006	0.132	0.133	2.370	0.680	
FCl···NH_3_ (C_3v_)	−10.13	−9.39	2.320	0.145	0.358	−0.058	0.311	0.216	2.687	0.729	
FSH···NH_3_ (C_s_)	−8.23	−7.58	2.512	0.081	0.235	−0.020	0.194	0.166	3.309	0.820	
FPH_2_···NH_3_ (C_s_)	−6.81	−6.10	2.663	0.057	0.171	−0.012	0.144	0.140	3.794	0.886	
FCl···Cl^−^ (C_*∞*v_)	−30.07	−28.98	2.316	0.496	0.547	−0.161	0.855	0.382	1.212	0.465	
FSH···Cl^−^ (C_s_)	−23.46	−22.48	2.493	0.305	0.377	−0.092	0.443	0.264	1.466	0.518	
FPH_2_···Cl^−^ (C_s_)	−19.62	−18.62	2.649	0.208	0.266	−0.058	0.307	0.214	2.136	0.641	

* Binding energies (ΔE) and conterpoise corrected binding energies Δ$E_{cp}$ in kcal/mol. Intermonomer bond distance r(TA) in Å. Density at the TA critical point $\rho_{b}$ in e/Å^3^, energy density at the TA critical point $H_{b}$ in Hartree/Å^3^. NPA charge transfer (CT) in e. TA and XT local stretching force constant ($k^{a}$) in mdyn/Å and BSO *n* values. Computed at the CCSD(T)/aug-cc-pVTZ level of theory.

## Supplementary Materials

The following are available online, Figure S1: Schematic representation of monomers (**36–63**) with atomic charges from the natural population analysis, Figure S2: Noncovalent interactions (NCIs) plot of complexes **1–35**, Figure S3: Selected molecular orbitals of the T-acceptors, Figure S4: Electron difference density distributions Δρ(r) for complexes **1–35**, Figure S5: Combination of donor and acceptor NBO orbitals involved in the electron delocalization of selected complexes; Table S1: Comparison between DLPNO-CCSD(T)/aug-cc-pV5Z and CCSD(T)/aug-cc-pVTZ energies, Table S2: Deviation from DLPNO-CCSD(T)/aug-cc-pV5Z interaction energies, Table S3: Atomic Cartesian coordinates in Å of complexes **1–35**.

## Abbreviations

The following abbreviations are used in this manuscript:

ACS	Adibatic connection scheme
BSO *n*	Bond strength order
CCSD(T)	Coupled cluster theory with singles, doubles, and perturbative triples
CT	Intermonomer charge transfer
EDG	Electron donating group
EWG	Electron withdrawing group
HOMO	Highest occupied molecular orbital
LUMO	Lowest unoccupied molecular orbital
NBO	Natural bond orbital
NCI	Noncovalent interaction
NPA	Natural population analysis
TB	Tetrel bond
