# Supplementary material for: Quantitative Assessment of Tetrel Bonding Utilizing Vibrational Spectroscopy
Source: Molecules. 2018 Oct 25;23(11):2763. doi: 10.3390/molecules23112763 (PMC6278569; doi:10.3390/molecules23112763)
Supplement: Supplementary file 1 [file molecules-23-02763-s001.pdf]

Supporting Information: Quantitative Assessment of Tetrel Bonding Utilizing Vibrational Spectroscopy

Daniel Sethio, Vytor Oliveira, Elfi Kraka*

Computational and Theoretical Chemistry Group (CATCO),

Department of Chemistry, Southern Methodist University,

3215 Daniel Avenue, Dallas, Texas 75275-0314, United States

E-mail: ekraka@gmail.com

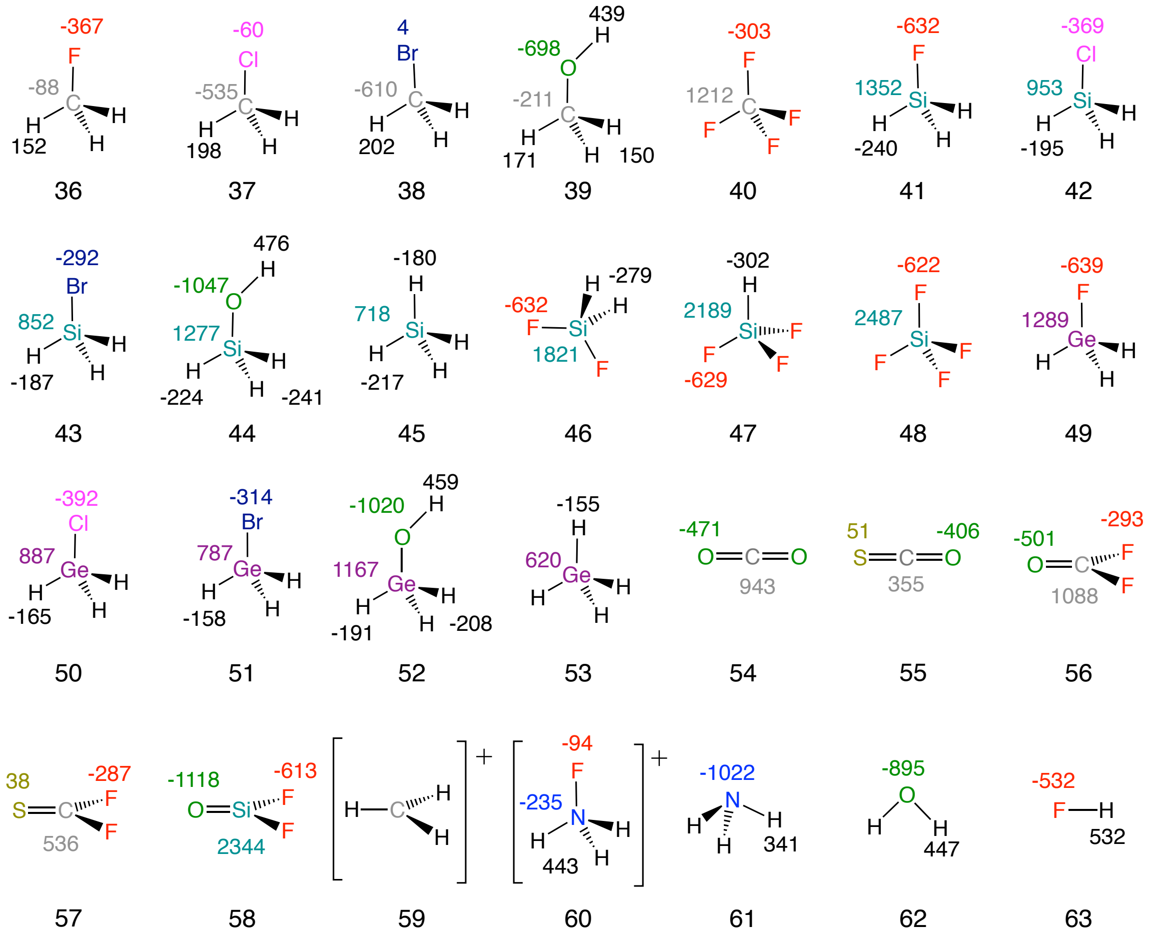

Figure S1: Schematic representation of monomers (**36-63**) with atomic charges from the natural population analysis calculated at CCSD(T)/aug-cc-pVTZ level of theory. Colors are used to correlate charges to specific atoms.

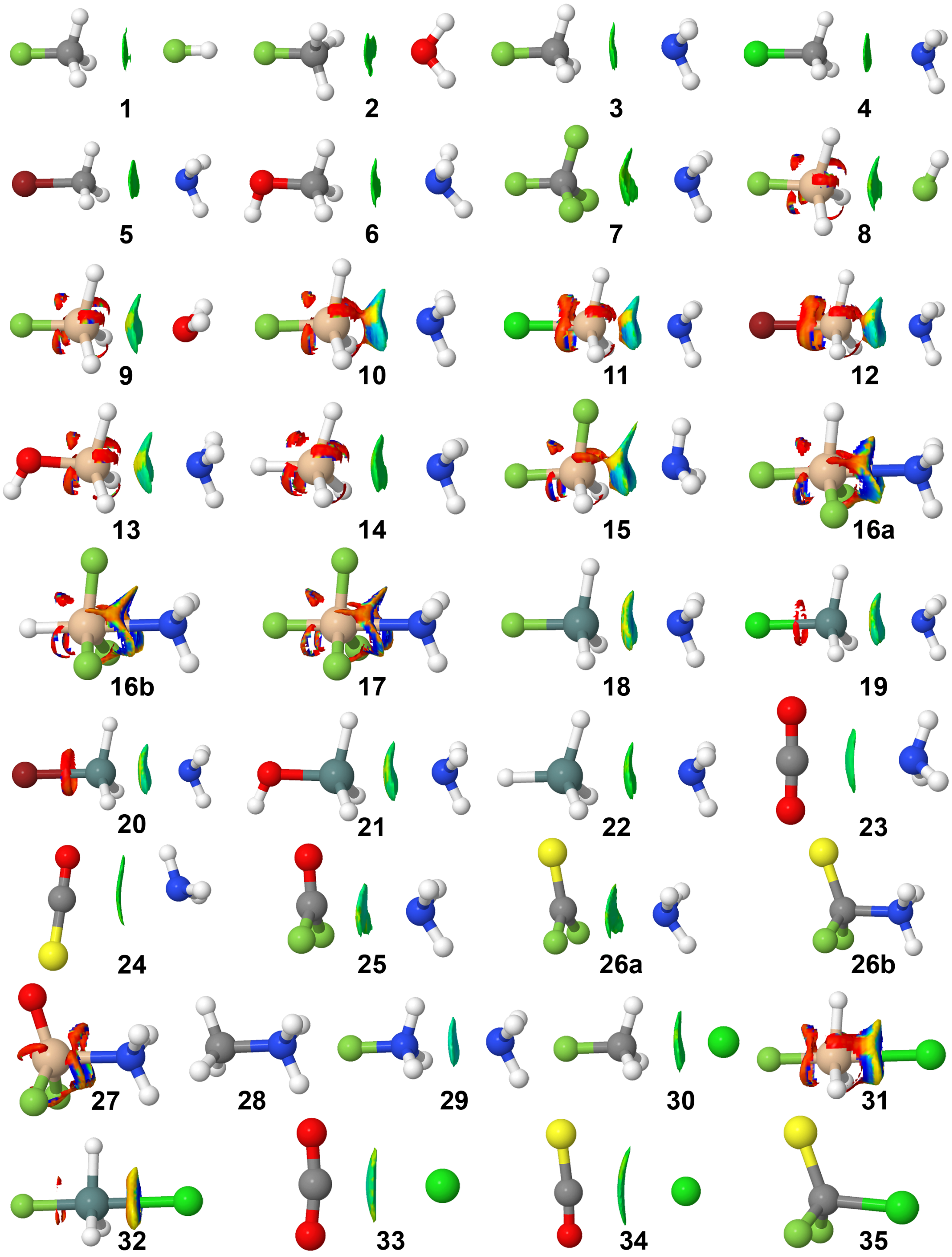

Figure S2: Noncovalent interactions (NCIs) plot of complexes **1-35** calculated at gradient isosurfaces $s = 0.5$ au., where green indicates weak attractive, blue indicates strong attractive, and red surface indicates repulsive noncovalent interactions.

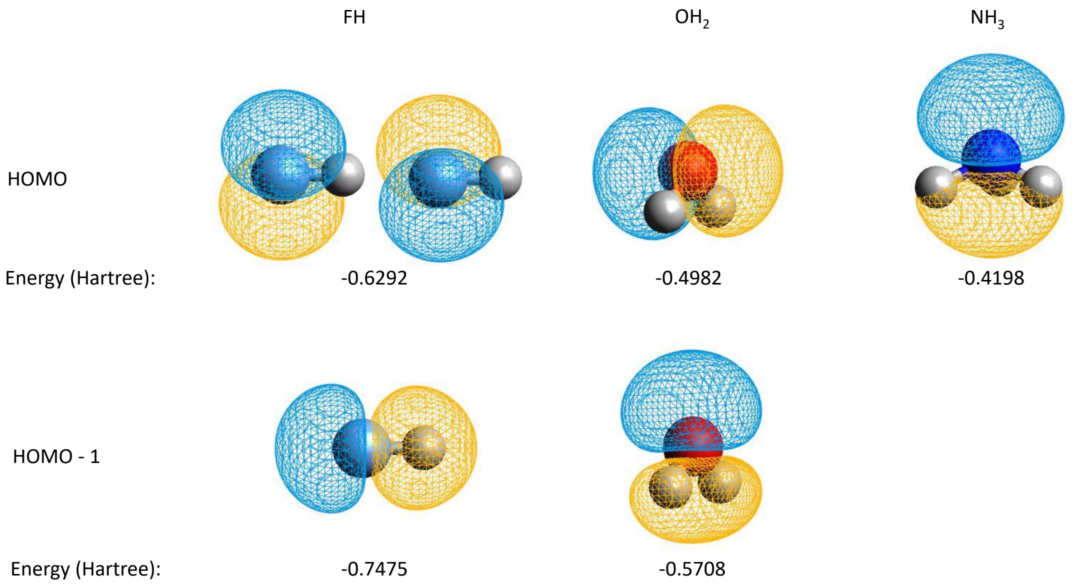

Figure S3: Selected molecular orbitals of the T-acceptors. Calculated at HF/6-31G(d) level of theory.

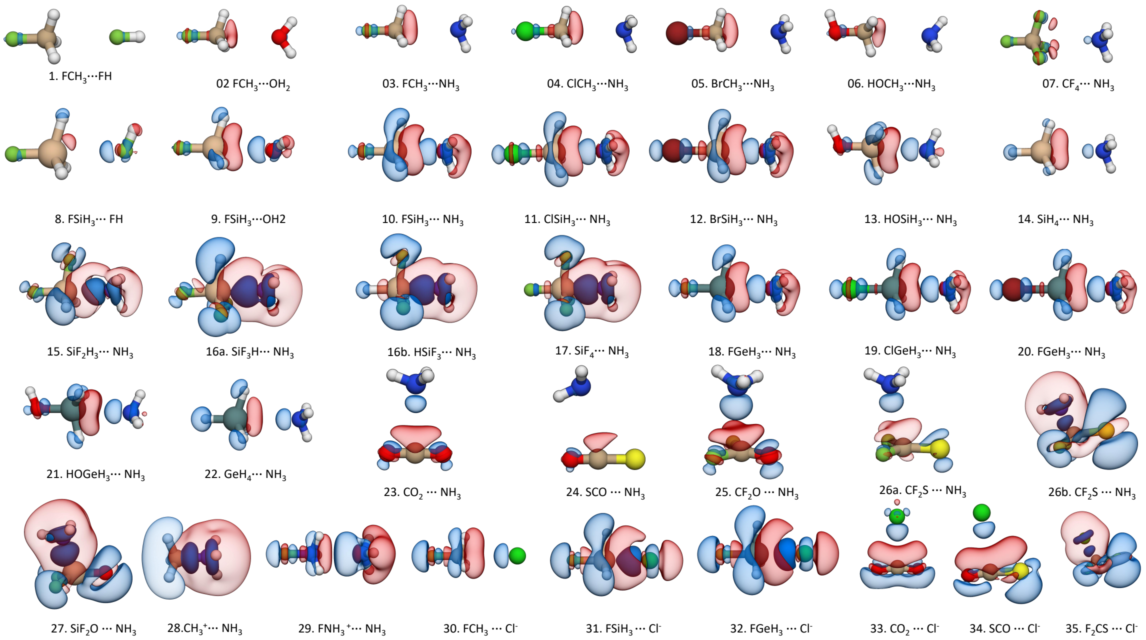

Figure S4: CCSD(T)/aug-cc-pVTZ electron difference density distributions $\Delta\rho(\mathbf{r})$ given for complexes **1-35**. $\Delta\rho(\mathbf{r})$ is plotted for an electron density surface with a constant density value of 0.001 a.u. Blue regions indicate an increase in the electron density, red regions indicate a density decrease relative to the superimposed density of the monomer.

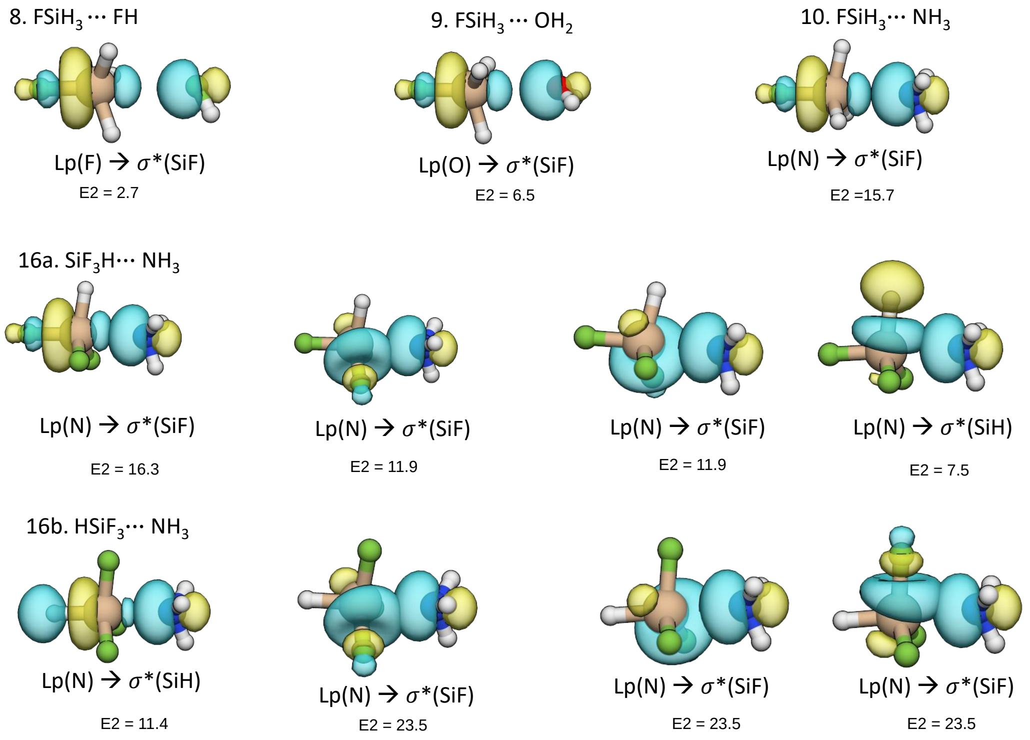

Figure S5: Combination of donor and acceptor NBO orbitals involved in the electron delocalization of selected complexes. Delocalization energies are given in kcal/mol

Table S1: Comparion between DLPNO-CCSD(T)/aug-cc-pV5Z and CCSD(T)/aug-cc-pVTZ energies*

#	Complex (symm.)	CCSD(T)/aug-cc-pVTZ E_{int}	E_{int}(CP)	BSSE	CCSD(T)/aug-cc-pV5Z E_{int}	E_{int}(CP)	BSSE
C - Tetrel bond							
**1**	$FCH_3 \cdots FH$ (C_{3v})	-1.51	-1.30	0.21	-1.36	-1.33	0.03
**2**	$FCH_3 \cdots OH_2$ (C_s)	-2.11	-1.89	0.22	-1.96	-1.93	0.03
**3**	$FCH_3 \cdots NH_3$ (C_{3v})	-2.27	-2.07	0.20	-2.15	-2.12	0.03
**4**	$ClCH_3 \cdots NH_3$ (C_{3v})	-2.09	-1.90	0.19	-2.00	-1.96	0.04
**5**	$BrCH_3 \cdots NH_3$ (C_{3v})	-2.02	-1.81	0.21	-1.91	-1.88	0.03
**6**	$HOCH_3 \cdots NH_3$ (C_s)	-1.39	-1.23	0.16	-1.27	-1.25	0.02
**7**	$CF_4 \cdots NH_3$ (C_{3v})	-1.69	-1.30	0.39	-1.38	-1.32	0.06
Si - Tetrel bond							
**8**	$FSiH_3 \cdots FH$ (C_s)	-2.34	-1.91	0.43	-2.08	-2.02	0.06
**9**	$FSiH_3 \cdots OH_2$ (C_s)	-4.55	-3.96	0.59	-4.31	-4.24	0.07
**10**	$FSiH_3 \cdots NH_3$ (C_{3v})	-8.91	-8.06	0.85	-8.81	-8.72	0.09
**11**	$ClSiH_3 \cdots NH_3$ (C_{3v})	-8.16	-7.44	0.72	-8.19	-8.09	0.10
**12**	$BrSiH_3 \cdots NH_3$ (C_{3v})	-8.35	-7.58	0.77	-8.39	-8.30	0.09
**13**	$HOSiH_3 \cdots NH_3$ (C_s)	-4.81	-4.29	0.52	-4.70	-4.64	0.06
**14**	$SiH_4 \cdots NH_3$ (C_{3v})	-2.41	-2.12	0.29	-2.32	-2.29	0.03
**15**	$SiF_2H_2 \cdots NH_3$ (C_s)	-11.73	-10.47	1.26	-11.46	-11.32	0.14
**16a**	$SiF_3H \cdots NH_3$ (C_s)	-19.43	-17.54	1.89	-19.06	-18.84	0.22
**16b**	$SiF_3H \cdots NH_3$ (C_{3v})	-27.53	-25.36	2.17	-27.29	-27.01	0.28
**17**	$SiF_4 \cdots NH_3$ (C_{3v})	-32.55	-30.00	2.55	-32.05	-31.75	0.30
Ge - Tetrel bond							
**18**	$FGeH_3 \cdots NH_3$ (C_{3v})	-9.17	-8.58	0.59	-9.04	-8.97	0.07
**19**	$ClGeH_3 \cdots NH_3$ (C_{3v})	-7.29	-6.82	0.47	-7.28	-7.20	0.08
**20**	$BrGeH_3 \cdots NH_3$ (C_{3v})	-7.08	-6.60	0.48	-7.09	-7.01	0.08
**21**	$HOGeH_3 \cdots NH_3$ (C_s)	-5.08	-4.68	0.40	-4.97	-4.92	0.05
**22**	$GeH_4 \cdots NH_3$ (C_{3v})	-2.08	-1.88	0.20	-2.01	-1.99	0.02
Double bond - Tetrel bond							
**23**	$CO_2 \cdots NH_3$ (C_s)	-3.21	-2.95	0.26	-3.09	-3.04	0.05
**24**	$SCO \cdots NH_3$ (C_s)	-1.99	-1.71	0.28	-1.79	-1.75	0.04
**25**	$CF_2O \cdots NH_3$ (C_s)	-5.81	-5.09	0.72	-5.35	-5.25	0.10
**26a**	$CF_2S \cdots NH_3$ (C_s)	-4.02	-3.34	0.68	-3.53	-3.44	0.09
**26b**	$CF_2S \cdots NH_3$ (C_s)	-22.68	-19.85	2.83	-23.73	-23.27	0.46
**27**	$SiF_2O \cdots NH_3$ (C_s)	-52.10	-50.12	1.98	-53.34	-53.07	0.27
Anionic - Tetrel bond							
**28**	$CH_3^+ \cdots NH_3$ (C_{3v})	-135.20	-133.96	1.24	-135.92	-135.74	0.18
**29**	$FNH_3^+ \cdots NH_3$ (C_{3v})	-23.56	-23.20	0.36	-23.49	-23.43	0.06
**30**	$FCH_3 \cdots Cl^-$ (C_{3v})	-10.16	-9.73	0.43	-10.11	-9.98	0.13
**31**	$FSiH_3 \cdots Cl^-$ (C_{3v})	-32.76	-31.52	1.24	-33.42	-33.14	0.28
**32**	$FGeH_3 \cdots Cl^-$ (C_{3v})	-36.81	-35.79	1.02	-37.18	-36.93	0.25
**33**	$CO_2 \cdots Cl^-$ (C_s)	-8.89	-8.43	0.46	-8.89	-8.77	0.12
**34**	$SCO \cdots Cl^-$ (C_s)	-5.88	-5.48	0.40	-5.85	-5.75	0.10
**35**	$CF_2S \cdots Cl^-$ (C_s)	-49.44	-46.46	2.98	-50.61	-50.02	0.59

*Interaction energies (E_{int}), counterpoise (CP) corrected interaction energies and basis set superposition error calculated at CCSD(T)/aug-cc-pVTZ and DLPNO-CCSD(T)/aug-cc-pV5Z levels. All energies were obtained using CCSD(T)/aug-cc-pVTZ geometries. Values are given in kcal/mol.

Table S2: Deviation from DLPNO-CCSD(T)/aug-cc-pV5Z interaction energies*

#	Complex (symm.)	(a)	(b)	(c)
C - Tetrel bond				
**1**	$FCH_3 \cdots FH$ (C_{3v})	-0.03	0.18	0.15
**2**	$FCH_3 \cdots OH_2$ (C_s)	-0.04	0.18	0.15
**3**	$FCH_3 \cdots NH_3$ (C_{3v})	-0.05	0.15	0.12
**4**	$ClCH_3 \cdots NH_3$ (C_{3v})	-0.06	0.13	0.09
**5**	$BrCH_3 \cdots NH_3$ (C_{3v})	-0.07	0.14	0.11
**6**	$HOCH_3 \cdots NH_3$ (C_s)	-0.02	0.14	0.12
**7**	$CF_4 \cdots NH_3$ (C_{3v})	-0.02	0.37	0.31
Si - Tetrel bond				
**8**	$FSiH_3 \cdots FH$ (C_s)	-0.11	0.32	0.26
**9**	$FSiH_3 \cdots OH_2$ (C_s)	-0.28	0.31	0.24
**10**	$FSiH_3 \cdots NH_3$ (C_{3v})	-0.66	0.19	0.10
**11**	$ClSiH_3 \cdots NH_3$ (C_{3v})	-0.65	0.07	-0.03
**12**	$BrSiH_3 \cdots NH_3$ (C_{3v})	-0.72	0.05	-0.04
**13**	$HOSiH_3 \cdots NH_3$ (C_s)	-0.35	0.17	0.11
**14**	$SiH_4 \cdots NH_3$ (C_{3v})	-0.17	0.12	0.09
**15**	$SiF_2H_2 \cdots NH_3$ (C_s)	-0.85	0.41	0.27
**16a**	$SiF_3H \cdots NH_3$ (C_s)	-1.30	0.59	0.37
**16b**	$SiF_3H \cdots NH_3$ (C_{3v})	-1.65	0.52	0.24
**17**	$SiF_4 \cdots NH_3$ (C_{3v})	-1.75	0.80	0.50
Ge - Tetrel bond				
**18**	$FGeH_3 \cdots NH_3$ (C_{3v})	-0.39	0.20	0.13
**19**	$ClGeH_3 \cdots NH_3$ (C_{3v})	-0.38	0.09	0.01
**20**	$BrGeH_3 \cdots NH_3$ (C_{3v})	-0.41	0.07	-0.01
**21**	$HOGeH_3 \cdots NH_3$ (C_s)	-0.24	0.16	0.11
**22**	$GeH_4 \cdots NH_3$ (C_{3v})	-0.11	0.09	0.07
Double bond - Tetrel bond				
**23**	$CO_2 \cdots NH_3$ (C_s)	-0.09	0.17	0.12
**24**	$SCO \cdots NH_3$ (C_s)	-0.04	0.24	0.20
**25**	$CF_2O \cdots NH_3$ (C_s)	-0.16	0.56	0.46
**26a**	$CF_2S \cdots NH_3$ (C_s)	0.10	-0.58	-0.49
**26b**	$CF_2S \cdots NH_3$ (C_s)	-3.42	-0.59	-1.05
**27**	$SiF_2O \cdots NH_3$ (C_s)	-2.95	-0.97	-1.24
Anionic - Tetrel bond				
**28**	$CH_3^+ \cdots NH_3$ (C_{3v})	-1.78	-0.54	-0.72
**29**	$FNH_3^+ \cdots NH_3$ (C_{3v})	-0.23	0.13	0.07
**30**	$FCH_3 \cdots Cl^-$ (C_{3v})	-0.25	0.18	0.05
**31**	$FSiH_3 \cdots Cl^-$ (C_{3v})	-1.62	-0.38	-0.66
**32**	$FGeH_3 \cdots Cl^-$ (C_{3v})	-1.14	-0.12	-0.37
**33**	$CO_2 \cdots Cl^-$ (C_s)	-0.34	0.12	0.00
**34**	$SCO \cdots Cl^-$ (C_s)	-0.27	0.13	0.03
**35**	$CF_2S \cdots Cl^-$ (C_s)	-3.56	-0.58	-1.17
	MAD	0.71	0.29	0.28

*Difference between DLPNO-CCSD(T)/aug-cc-pV5Z $E_{int}(CP)$ and CCSD(T)/aug-cc-pVTZ $E_{int}(CP)$ (a) and CCSD(T)/aug-cc-pVTZ E_{int} (b). Difference between DLPNO-CCSD(T)/aug-cc-pVTZ E_{int} and CCSD(T)/aug-cc-pVTZ E_{int} (c). Energies and mean absolute deviation (MAD) given in kcal/mol.

Table S3: Atomic Cartesian coordinates (in Å) of complexes 1-35 optimized at the CCSD(T)/aug-cc-pVTZ level of theory.

1 $FCH_3\cdots FH$, C_{3v}

C	-0.64760	0.00000	0.00000
F	-2.03974	0.00000	0.00000
H	-0.29694	0.51589	-0.89355
H	-0.29694	-1.03178	0.00000
H	-0.29694	0.51589	0.89355
F	2.32388	0.00000	0.00000
H	3.24545	0.00000	0.00000

2 $FCH_3\cdots OH_2$, C_s

C	0.58503	-0.01958	0.00000
F	1.97844	0.01163	0.00000
H	0.25798	-1.05889	0.00000
H	0.22171	0.48606	-0.89362
H	0.22171	0.48606	0.89362
O	-2.44987	0.00395	0.00000
H	-3.06002	-0.73963	0.00000
H	-3.02146	0.77758	0.00000

3 $FCH_3\cdots NH_3$, C_{3v}

C	-0.59814	0.00000	0.00000
F	-1.99271	0.00000	0.00000
H	-0.24618	-0.51535	-0.89263
H	-0.24618	1.03070	0.00000

H	-0.24618	-0.51535	0.89263
N	2.61945	0.00000	0.00000
H	3.00978	0.46868	0.81177
H	3.00978	0.46868	-0.81177
H	3.00978	-0.93735	0.00000

**4**	$ClCH_3 \cdots NH_3$, C_{3v}		
C	0.06943	0.00000	0.00000
Cl	-1.72827	0.00000	0.00000
H	0.41195	-0.51492	-0.89188
H	0.41195	1.02985	0.00000
H	0.41195	-0.51492	0.89188
N	3.35811	0.00000	0.00000
H	3.74840	0.46869	0.81179
H	3.74840	0.46869	-0.81179
H	3.74840	-0.93738	0.00000

**5**	$BrCH_3 \cdots NH_3$, C_{3v}		
C	0.86226	0.00000	0.00000
Br	-1.09071	0.00000	0.00000
H	1.19569	-0.51600	-0.89374
H	1.19569	1.03200	0.00000
H	1.19569	-0.51600	0.89374
N	4.16614	0.00000	0.00000
H	4.55633	0.46871	0.81183
H	4.55633	0.46871	-0.81183

H	4.55633	-0.93742	0.00000

**6**	(OH)CH_3···NH_3, C_s		
H	-2.49864	0.81659	0.00000
O	-2.13732	-0.07379	0.00000
C	-0.71198	0.03232	0.00000
H	-0.32011	-0.98345	0.00000
H	-0.33794	0.54691	0.88947
H	-0.33794	0.54691	-0.88947
N	2.65001	0.01052	0.00000
H	3.28000	0.80658	0.00000
H	2.89631	-0.54671	-0.81216
H	2.89631	-0.54671	0.81216

**7**	CF_4···NH_3, C_{3v}		
C	-0.55900	0.00000	0.00000
F	-1.88668	0.00000	0.00000
F	-0.13050	-0.62387	-1.08058
F	-0.13050	1.24774	0.00000
F	-0.13050	-0.62387	1.08058
N	2.86699	0.00000	0.00000
H	3.25551	0.46896	0.81227
H	3.25551	0.46896	-0.81227
H	3.25551	-0.93793	0.00000

8 $FSiH_3$···FH, C_s

Si	0.43320	0.01974	0.00000
F	2.04988	-0.02546	0.00000
H	-0.03966	-1.38225	0.00000
H	0.00693	0.72855	-1.22281
H	0.00693	0.72855	1.22281
F	-2.53084	0.03461	0.00000
H	-2.93319	-0.79531	0.00000

**9**	$FSiH_3 \cdots OH_2$, C_s		
Si	0.01186	0.31211	0.00000
F	-0.00996	1.93530	0.00000
H	1.43780	-0.07630	0.00000
H	-0.68509	-0.11005	-1.23076
H	-0.68509	-0.11005	1.23076
O	-0.05586	-2.46085	0.00000
H	0.33866	-2.89719	-0.76172
H	0.33866	-2.89719	0.76172

**10**	$FSiH_3 \cdots NH_3$, C_{3v}		
Si	-0.20928	0.00000	0.00000
F	-1.84579	0.00000	0.00000
H	0.12338	-0.72003	-1.24713
H	0.12338	1.44006	0.00000
H	0.12338	-0.72003	1.24713
N	2.31409	0.00000	0.00000
H	2.69382	0.47094	0.81569

H	2.69382	0.47094	-0.81569
H	2.69382	-0.94188	0.00000

11 $ClSiH_3 \cdots NH_3$, C_{3v}

Si	0.33660	0.00000	0.00000
Cl	-1.78021	0.00000	0.00000
H	0.66936	-0.71839	-1.24428
H	0.66936	1.43677	0.00000
H	0.66936	-0.71839	1.24428
N	2.91649	0.00000	0.00000
H	3.29796	0.47068	0.81525
H	3.29796	0.47068	-0.81525
H	3.29796	-0.94136	0.00000

12 $BrSiH_3 \cdots NH_3$, C_{3v}

Si	1.06281	0.00000	0.00000
Br	-1.22723	0.00000	0.00000
H	1.38429	-0.71948	-1.24615
H	1.38429	1.43893	0.00000
H	1.38429	-0.71948	1.24615
N	3.62836	0.00000	0.00000
H	4.00960	0.47077	0.81539
H	4.00960	0.47077	-0.81539
H	4.00960	-0.94154	0.00000

13 $(HO)SiH_3 \cdots NH_3$, C_s

H	-2.48714	0.75291	0.00000
O	-2.00799	-0.07791	0.00000
Si	-0.33120	0.02073	0.00000
H	0.07573	-1.39753	0.00000
H	0.10142	0.74724	1.21938
H	0.10142	0.74724	-1.21938
N	2.49327	-0.00992	0.00000
H	2.89338	0.92319	0.00000
H	2.86755	-0.48701	-0.81436
H	2.86755	-0.48701	0.81436

14 $SiH_4 \cdots NH_3$, C_{3v}

Si	-1.13339	0.00000	0.00000
H	-2.62311	0.00000	0.00000
H	-0.67520	-0.70437	-1.22000
H	-0.67520	1.40873	0.00000
H	-0.67520	-0.70437	1.22000
N	2.06884	0.00000	0.00000
H	2.45533	0.46945	0.81310
H	2.45533	0.46945	-0.81310
H	2.45533	-0.93887	0.00000

15 $SiF_2H_2 \cdots NH_3$, C_s

F	-0.12376	1.22628	0.00000
Si	0.25601	-0.33778	0.00000
F	1.87344	-0.28456	0.00000

H	-0.05846	-1.00506	1.26959
H	-0.05846	-1.00506	-1.26959
N	-2.29934	-0.38588	0.00000
H	-2.54738	0.59923	0.00000
H	-2.73886	-0.80171	-0.81548
H	-2.73886	-0.80171	0.81548

16a $SiF_3H{\cdots}NH_3$, C_s

H	1.75218	-0.00856	0.00000
Si	0.29667	0.17654	0.00000
F	0.24053	1.79282	0.00000
F	-0.52830	-0.08799	-1.35509
F	-0.52830	-0.08799	1.35509
N	0.32143	-2.02817	0.00000
H	-0.63431	-2.37362	0.00000
H	0.78216	-2.40878	0.82139
H	0.78216	-2.40878	-0.82139

16b $HSiF_3{\cdots}NH_3$, C_{3v}

Si	0.47664	0.00000	0.00000
H	1.95077	0.00000	0.00000
F	0.23699	0.80167	-1.38853
F	0.23699	-1.60333	0.00000
F	0.23699	0.80167	1.38853
N	-1.62736	0.00000	0.00000
H	-1.99087	-0.47405	0.82109

H	-1.99087	-0.47405	-0.82109
H	-1.99087	0.94811	0.00000

17 $SiF_4 \cdots NH_3$, C_{3v}

Si	-0.14293	0.00000	0.00000
F	-1.75198	0.00000	0.00000
F	0.05853	-0.79697	-1.38040
F	0.05853	1.59395	0.00000
F	0.05853	-0.79697	1.38040
N	1.92903	0.00000	0.00000
H	2.29379	0.47403	0.82104
H	2.29379	0.47403	-0.82104
H	2.29379	-0.94806	0.00000

18 $FGeH_3 \cdots NH_3$, C_{3v}

Ge	-0.10836	0.00000	0.00000
F	-1.92384	0.00000	0.00000
H	0.18762	-0.75421	-1.30632
H	0.18762	1.50841	0.00000
H	0.18762	-0.75421	1.30632
N	2.51587	0.00000	0.00000
H	2.89814	0.47046	0.81486
H	2.89814	0.47046	-0.81486
H	2.89814	-0.94092	0.00000

19 $ClGeH_3 \cdots NH_3$, C_{3v}

Ge	0.21998	0.00000	0.00000
Cl	-1.99578	0.00000	0.00000
H	0.56785	-0.74722	-1.29422
H	0.56785	1.49443	0.00000
H	0.56785	-0.74722	1.29422
N	2.97467	0.00000	0.00000
H	3.35953	0.46997	0.81402
H	3.35953	0.46997	-0.81402
H	3.35953	-0.93995	0.00000

20 $BrGeH_3 \cdots NH_3$, C_{3v}

Ge	0.79891	0.00000	0.00000
Br	-1.57624	0.00000	0.00000
H	1.14870	-0.74687	-1.29360
H	1.14870	1.49372	0.00000
H	1.14870	-0.74687	1.29360
N	3.56508	0.00000	0.00000
H	3.94994	0.46998	0.81403
H	3.94994	0.46998	-0.81403
H	3.94994	-0.93997	0.00000

21 $(HO)GeH_3 \cdots NH_3$, C_s

H	-2.43370	0.76387	0.00000
O	-2.00116	-0.09434	0.00000
Ge	-0.18595	0.01398	0.00000
H	0.20520	-1.47028	0.00000

H	0.22916	0.77228	1.27946
H	0.22916	0.77228	-1.27946
N	2.72363	-0.01997	0.00000
H	3.13198	0.90970	0.00000
H	3.09702	-0.49920	-0.81368
H	3.09702	-0.49920	0.81368

22 $GeH_4 \cdots NH_3$, C_{3v}

Ge	-0.60690	0.00000	0.00000
H	-2.15735	0.00000	0.00000
H	-0.12312	-0.73139	-1.26680
H	-0.12312	1.46278	0.00000
H	-0.12312	-0.73139	1.26680
N	2.71572	0.00000	0.00000
H	3.10263	0.46934	0.81291
H	3.10263	0.46934	-0.81291
H	3.10263	-0.93867	0.00000

23 $CO_2 \cdots NH_3$, C_s

H	-2.46031	0.93617	0.00000
N	-2.10155	-0.01349	0.00000
C	0.82078	0.00415	0.00000
O	0.83040	1.17131	0.00000
O	0.86401	-1.16202	0.00000
H	-2.50221	-0.47280	-0.81190
H	-2.50221	-0.47280	0.81190

24 SCO···NH_3, C_s

H	-2.74176	0.25897	0.00000
N	-2.51252	-0.73021	0.00000
C	0.40035	0.61529	0.00000
O	-0.30293	1.54197	0.00000
S	1.37539	-0.61935	0.00000
H	-2.97013	-1.13168	-0.81234
H	-2.97013	-1.13168	0.81234

25 CF_2O···NH_3, C_s

N	-2.13545	0.02830	0.00000
C	0.54866	0.15032	0.00000
O	0.63058	1.32554	0.00000
F	0.54839	-0.62376	-1.06358
F	0.54839	-0.62376	1.06358
H	-2.68397	-0.82584	0.00000
H	-2.43069	0.56117	-0.81244
H	-2.43069	0.56117	0.81244

26a CF_2S···NH_3, C_s

N	-2.24144	1.00020	0.00000
C	0.28875	-0.41164	0.00000
S	1.33700	0.80678	0.00000
F	-0.18946	-1.01830	-1.05969
F	-0.18946	-1.01830	1.05969

H	-3.16833	0.58588	0.00000
H	-2.19908	1.60796	-0.81245
H	-2.19908	1.60796	0.81245

26b $CF_2S{\cdots}NH_3$, C_s

N	-0.81775	1.37793	0.00000
C	-0.18921	-0.07947	0.00000
S	1.51010	0.00257	0.00000
F	-0.83680	-0.61886	-1.08325
F	-0.83680	-0.61886	1.08325
H	-1.83974	1.36073	0.00000
H	-0.45152	1.84535	-0.82816
H	-0.45152	1.84535	0.82816

27 $SiF_2O{\cdots}NH_3$, C_s

N	-0.42376	1.66094	0.00000
Si	0.20327	-0.15088	0.00000
O	1.73129	-0.09429	0.00000
F	-0.68147	-0.62119	-1.25214
F	-0.68147	-0.62119	1.25214
H	-1.43724	1.75497	0.00000
H	-0.05066	2.13614	-0.81948
H	-0.05066	2.13614	0.81948

28 $CH_3^+{\cdots}NH_3$, C_{3v}

N	0.70513	0.00000	0.00000

C	-0.80599	0.00000	0.00000
H	-1.14296	0.51675	-0.89504
H	-1.14296	-1.03351	0.00000
H	-1.14296	0.51675	0.89504
H	1.07613	-0.47653	0.82539
H	1.07613	-0.47653	-0.82539
H	1.07613	0.95307	0.00000

29 $FNH_3^+ \cdots NH_3$, C_{3v}

N	-0.39134	0.00000	0.00000
F	-1.76513	0.00000	0.00000
H	-0.05710	-0.48443	-0.83905
H	-0.05710	0.96885	0.00000
H	-0.05710	-0.48443	0.83905
N	2.22808	0.00000	0.00000
H	2.64176	0.46550	0.80625
H	2.64176	0.46550	-0.80625
H	2.64176	-0.93099	0.00000

30 $FCH_3 \cdots Cl^-$, C_{3v}

C	-1.23588	0.00000	0.00000
F	-2.65510	0.00000	0.00000
H	-0.88447	-0.51407	-0.89040
H	-0.88447	1.02814	0.00000
H	-0.88447	-0.51407	0.89040
Cl	1.94309	0.00000	0.00000

31 $FSiH_3 \cdots Cl^-$, C_{3v}

Si	-0.65514	0.00000	0.00000
F	-2.35776	0.00000	0.00000
H	-0.50131	-0.73709	-1.27667
H	-0.50131	1.47418	0.00000
H	-0.50131	-0.73709	1.27667
Cl	1.84845	0.00000	0.00000

32 $FGeH_3 \cdots Cl^-$, C_{3v}

Ge	-0.41308	0.00000	0.00000
F	-2.30517	0.00000	0.00000
H	-0.31390	-0.76825	-1.33064
H	-0.31390	1.53649	0.00000
H	-0.31390	-0.76825	1.33064
Cl	2.15274	0.00000	0.00000

33 $CO_2 \cdots Cl^-$, C_s

Cl	0.00000	0.00000	1.66499
C	0.00000	0.00000	-1.25540
O	0.00000	1.16587	-1.34912
O	0.00000	-1.16587	-1.34912

34 $SCO \cdots Cl^-$, C_s

Cl	2.05468	0.23409	0.00000
C	-0.96955	-0.62147	0.00000

O	-0.65121	-1.73711	0.00000
S	-1.55759	0.84626	0.00000

35 $CF_2S \cdots Cl^-$, C_s

Cl	-1.49219	0.66590	0.00000
C	0.15369	-0.27857	0.00000
S	1.57057	0.70491	0.00000
F	0.00320	-1.11800	-1.07951
F	0.00320	-1.11800	1.07951
